# Selective Photochemical Oxidation of Reduced Dissolved Organic Sulfur to Inorganic Sulfate

**DOI:** 10.1021/acs.estlett.3c00210

**Published:** 2023-05-03

**Authors:** Brett A. Poulin

**Affiliations:** † Department of Environmental Toxicology, University of California Davis, Davis, California 95616, United States

**Keywords:** Dissolved organic sulfur, Desulfurization, DOM photochemistry, S-XANES

## Abstract

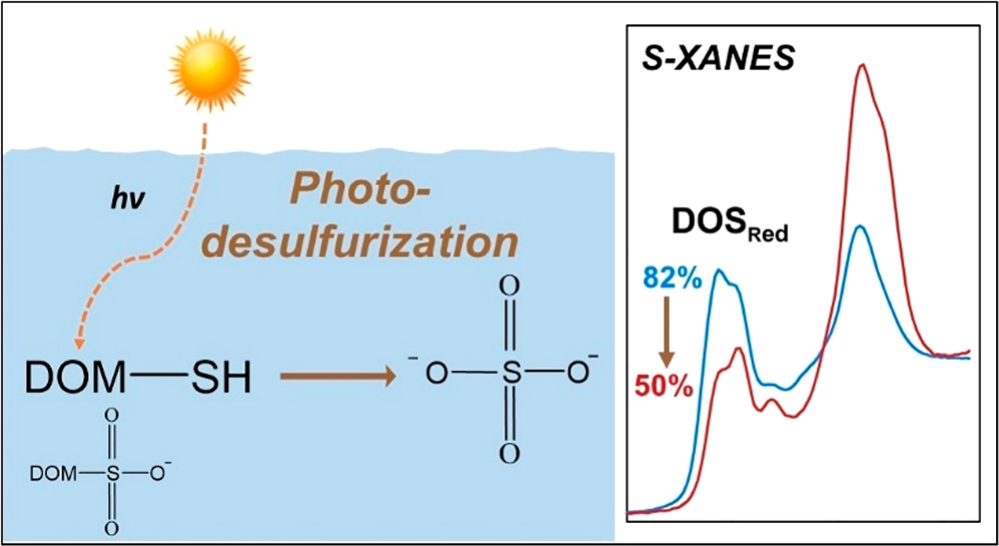

The chemical nature and stability of reduced dissolved organic sulfur (DOS_Red_) have implications on the biogeochemical cycling of trace and major elements across fresh and marine aquatic environments, but the underlying processes governing DOS_Red_ stability remain obscure. Here, dissolved organic matter (DOM) was isolated from a sulfidic wetland, and laboratory experiments quantified dark and photochemical oxidation of DOS_Red_ using atomic-level measurement of sulfur X-ray absorption near-edge structure (XANES) spectroscopy. DOS_Red_ was completely resistant to oxidation by molecular oxygen in the dark and underwent rapid and quantitative oxidation to inorganic sulfate (SO_4_^2–^) in the presence of sunlight. The rate of DOS_Red_ oxidation to SO_4_^2–^ greatly exceeded that of DOM photomineralization, resulting in a 50% loss of total DOS and 78% loss of DOS_Red_ over 192 h of irradiance. Sulfonates (DOS_SO3_) and other minor oxidized DOS functionalities were not susceptible to photochemical oxidation. The observed susceptibility of DOS_Red_ to photodesulfurization, which has implications on carbon, sulfur, and mercury cycling, should be comprehensively evaluated across diverse aquatic environments of differing DOM composition.

## Introduction

Dissolved organic sulfur (DOS) is a dynamic constituent of fresh^1^ and marine waters^2,3^ that influences diverse biogeochemical processes, including the cycling of sulfur (S) between organic and inorganic forms,^1^ formation of atmospheric organic S species (e.g., carbonyl sulfide (COS), carbonyl disulfide (CS_2_), and dimethyl sulfide (DMS)),^4^ and transport,^5^ bioavailability,^6^ and photochemical reactivity of mercury (Hg).^7,8^ The abiotic sulfurization of dissolved organic matter (DOM), involving nucleophilic addition of inorganic sulfide into DOM as reduced DOS (DOS_Red_) (namely, thiols),^1,3,9^ occurs in wastewater treatment systems,^10^ wetlands^1^ and estuaries,^11^ sulfidic lakes,^12^ and diverse marine waters.^13−15^ Aside from thiols, DOS_Red_ can be present as thioethers, disulfides, and perhaps thiophenes.^3,16^ Low-molecular-weight thiols (e.g., cysteine, glutathione) rapidly oxidize in dark and light oxic conditions,^17,18^ whereas DOS_Red_ is abundant, ranging from 50 to 70% of total DOS in freshwaters.^1,16,19^ Concentrations of DOS_Red_^1^ exceed low-molecular-weight thiols by 2–3 orders of magnitude.^20^ Therefore, understanding the stability of DOS is central to ascertaining the implications of DOS chemistry on the above-mentioned biogeochemical processes. To date, no studies have quantified the atomic-level transformation of DOS_Red_ due to dark and light oxidation, as research has probed photochemical changes in low-molecular-weight thiols^17,21,22^ or total DOS loss,^23−25^ or formation of organic and inorganic byproducts.^4,21^

Here, the stability of DOS_Red_ to light and dark oxidation was quantified by atomic measurements of S X-ray absorption spectroscopy, which quantifies different DOS oxidation states. DOM was isolated from a sulfidic wetland known to have high DOS_Red_^1^ and subjected to laboratory oxidation by O_2_ in the dark and artificial sunlight. Quantified changes in the DOM S content, DOS oxidation states, and inorganic S byproducts provide a more complete assessment of the stability of DOS_Red_ in aquatic environments.

## Materials and Methods

### DOM Sample Collection and Extraction

DOM was isolated from a representative sulfidic freshwater environment for laboratory experimentation as shown in Figure S1 and detailed in Section S1 of the Supporting Information (SI). Briefly, pore water was collected from a sulfidic Florida Everglades wetland (site WCA 2A-O; 26.42506°N, −80.47601°W) where DOS_Red_ is elevated,^1^ stored under N_2_ at 4 °C, and shipped on ice to the U.S. Geological Survey (Boulder, Colorado) for DOM isolation. The pore water was characterized in the field for pH (6.62), oxidation–reduction potential (−252 mV), dissolved oxygen (0.11 mg L^–1^), and sulfide (0.22 mM), and via laboratory measurements of dissolved organic carbon (DOC) (42.7 mgC L^–1^) and sulfate concentration (0.38 mM), and DOM specific ultraviolet absorbance at 254 nm (SUVA_254_) (3.4 L (mg m)^−1^).^26^ In the laboratory, residual inorganic sulfide was removed by purging with helium at pH 4.0, and the hydrophobic organic acid (HPOA) fraction of DOM was isolated on XAD-8 resin^27^ using trace-metal grade acids, degassed solutions, and N_2_-flushed tubing. An experiment (outlined in Section S2 of the SI) evaluated the oxidation of DOS during the elution step by comparing DOM isolated by XAD-8 resin (base elution) to PPL resin (methanol elution)^28^ and verified that DOM isolated by XAD-8 resin using deaerated solutions did not result in measurable oxidation of DOS (Figure S2, Tables S1–S2). The HPOA fraction accounted for 54% of the whole water DOC and was stored for up to 21 days (pH 3.5, under N_2_, 4 °C) for use in laboratory experiments.

### Laboratory Oxidation Experiments

The purified DOM sample was diluted with deaerated high-purity water (≥18 MΩ cm; Barnstead GenPro UV) to a DOC concentration of 37.9 mgC L^–1^ (pH 7) (complete details in SI and Figure S1), similar to surface waters of sulfate-enriched wetlands^1,12,26^ but lower than those in previous DOS photolysis studies.^21^ Although no pH buffer was used, subtle changes in pH expected from light exposure^29^ were not expected to dramatically influence the DOS photochemical oxidation rates.^17^ The initial DOM was sampled for characterization (termed t = 0 (Initial)). The following three experimental treatments were performed in 2 L quartz round-bottom flasks with 1 L of DOM solution (Figure S3a); the large volume was necessary for DOS characterization with this technique.^1^ (1) A dark anoxic control treatment (*n* = 1, termed Dark, Anoxic Control) was stored in the dark, under N_2_ at 22 ± 2 °C for 14 d to identify changes in DOS during storage or DOM reisolation. (2) A dark O_2_ purge treatment (*n* = 1, termed Dark, O_2_ Purge) quantified oxidation of DOS by O_2_ and was purged in the dark with zero-grade air for 192 h (20.5% O_2_, 79.5% N_2_; 30 mL min^–1^; 22 ± 2 °C). (3) The light treatment (termed Light 1–5 with one data point collected in duplicate, *n* = 6) was performed in a temperature-controlled solar simulator (Suntest XLS) at 500 W m^–2^ and 30 °C (300–800 nm irradiance range, spectrum provided in Figure S3b). Immediately before irradiation, DOM solutions were oxygen-saturated by purging with zero-grade air (98% saturation; an Orion RDO optical probe). Independent vessels were irradiated for 1.3, 5.3, 24 (*n* = 2), 78, and 192 h. Light treatments of 24–192 h duration were purged with zero-grade air every 12 h to prevent the depletion of O_2_.^21^

Following dark and light oxidation experiments, experimental solutions were sampled for aqueous measurements including DOC concentration, DOM absorption, and fluorescence properties (decadic absorption coefficients at 254 nm (α_254_) and 400 nm (α_400_); SUVA_254_;^30^ spectral slope from 275 to 295 nm (*S*_*275–295*_; x10^–3^ nm^–1^);^31^ humification index (HIX)^32^), and sulfate (SO_4_^2–^) and thiosulfate (S_2_O_3_^2–^) concentration by ion chromatography. Complete information on these measurements is provided in Section S1 of the SI. DOM optical measurements were used to identify changes in the DOM composition.^33^ Next, DOM solutions were deaerated and DOM was reisolated on XAD-8 resin (to remove inorganic S species), lyophilized, and stored under N_2_ for DOS characterization.

### DOS Characterization

Atomic S and C contents (and thus atomic S/C) of freeze-dried DOM samples were determined by Huffman Hazen Laboratories (Golden, CO) using International Humic Substances Society (IHSS) methods. Sulfur K-edge XANES spectra were collected on freeze-dried DOM samples (pressed as 5 mm pellets; see evaluation in Figure S4) on beamline 9-BM-B of the Advanced Photon Source (Argonne National Laboratory) as detailed previously^1^ and in Section S1e. DOS atomic fractions (*f*_DOS_X__) were determined with a precision estimated at ≤1.6% (based on measurement of IHSS samples)^16^ for exocyclic reduced (DOS_Exo_), heterocyclic reduced (DOS_Hetero_), sulfoxide (DOS_Sulfx_), sulfone (DOS_SO2_), sulfonate (DOS_SO3_), and organosulfate (DOS_SO4_). Nominal energies of DOS_Exo_ and DOS_Hetero_ are 2473.1 and 2474.4 eV, respectively, based on measurement of diverse model compounds.^16^ However, S-XANES spectra of reduced S model compounds likely in DOM (e.g., thiols, thioethers, disulfide, and thiophenes) span the energy range of DOS_Exo_ and DOS_Hetero_ (Figure S5)^16^ and cannot easily be resolved due to X-ray absorption doublets and shoulders. Thus, this study presents the total reduced DOS_Red_ defined in eq 1.
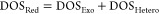
1Concentrations of DOS functionalities, relative to carbon, were calculated by multiplying the fraction of each DOS functionality by the atomic S/C. Aqueous concentrations of DOS functionalities ([DOS_X_]) were calculated using eq 2, where [DOC] is the DOC concentration measured on experimental solutions before DOM reisolation and the atomic S/C and *f*_DOS_X__ are measured on DOM extracts.
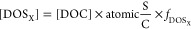
2Total S concentration (S_Tot_) was determined using eq 3, where [DOC] and [SO_4_^2–^] are the DOC and SO_4_^2–^ concentrations measured on experimental solutions before DOM reisolation, respectively, and the atomic S/C is of the DOM extract.
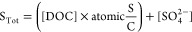
3

## Results and Discussion

### Dark Stability of Dissolved Organic Sulfur

**Figure 1:**
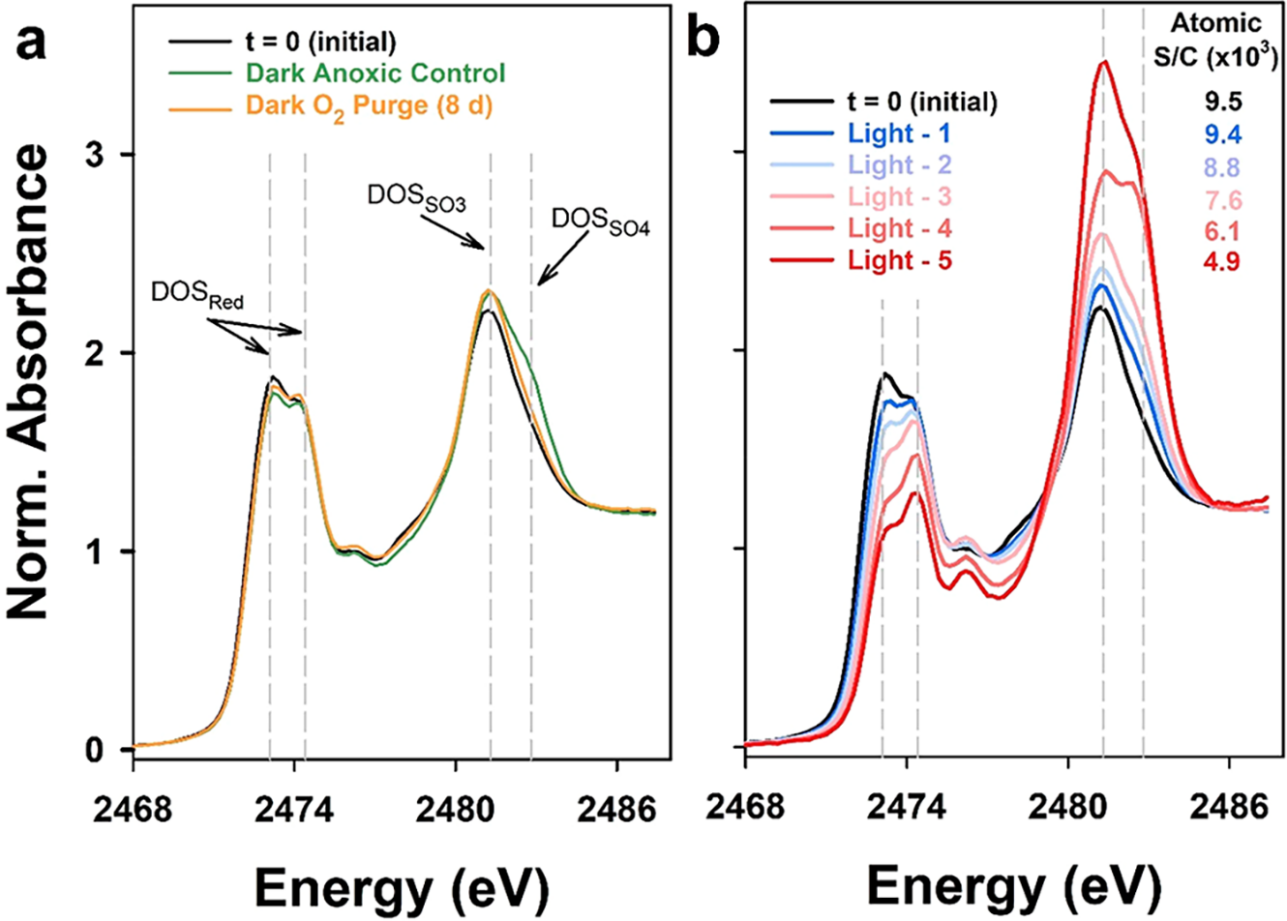
Sulfur K-edge XANES spectra comparing the DOM at the start of the experiment (*t* = 0 (initial)) to (a) the Dark Anoxic control and Dark O_2_ Purge (192 h) treatments and (b) light treatment (Light 1–5, 1.3–192 h). Gray dashed vertical lines identify nominal energies of DOS_Red_ (E_0_ = 2473.1 and 2474.4 eV for exocyclic and heterocyclic reduced S), sulfonate (DOS_SO3_, E_0_ = 2481.3 eV), and organosulfate (DOS_SO4_, E_0_ = 2482.8 eV) functionalities. In subplot a, spectra show no considerable change in DOM sulfur functionalities in the Dark Anoxic control and Dark O_2_ Purge (192 h) treatments compared to the initial sample (*t* = 0). In subplot b, spectra show a systematic decrease in the relative distribution of DOS_Red_ functionalities and an increase in the relative distribution of DOS_SO3_ and DOS_SO4_ with increased cumulative irradiance. The decrease in the relative distribution of DOS_Red_ functionalities is accompanied by a decrease in the atomic sulfur-to-carbon content (Atomic S/C) of the DOM. Gaussian decompositions of spectra and parameter values are provided in Figures S6 and S8 and Tables S3–S4.

DOM at the start of the experiment (t = 0 (initial)) showed elevated organic S content (atomic S/C = 9.6 × 10^–3^; Table 1) and an S K-edge XANES spectrum (Figure 1a) with prominent absorption at energies of DOS_Red_ functionalities. The distribution of DOS functionalities, based on spectral fitting (Figure S6, Table S3), quantified that DOS_Red_ accounted for 82% of total DOS in the t = 0 (initial) sample. Of the 82% of DOS_Red_, approximately two-thirds was highly reduced DOS_Exo_ and one-third was DOS_Hetero_. The concentration of DOS_Red_ in experimental solutions was 24.6 μM, whereas inorganic SO_4_^2–^ and S_2_O_3_^2–^ were minor components (1.0 μM and <0.45 μM, respectively). The high proportion of DOS_Red_ is consistent with previous investigations of sulfur-enriched Everglades wetlands^1,34^ and peat that has undergone sulfurization^35^ but higher than surface water DOM samples.^16^ Previous measurements of DOM from this location concluded that abiotic sulfurization yields DOS_Red_ primarily as thiols and thioethers, based on complementary use of S K-edge XANES spectroscopy and ultrahigh resolution mass spectrometry.^1^ Here, DOS_Red_ stability to dark oxidation was first evaluated under anoxic conditions (Dark Anoxic Control treatment) and by O_2_ (Dark O_2_ Purge treatment). The Dark Anoxic Control treatment, held anoxic for 14 days, exhibited minor differences in DOS content and functionality (atomic S/C = 9.7 × 10^–3^ and DOS_Red_ = 77% of total DOS, respectively) compared to the t = 0 (initial) sample (Figure 1a, Table 1); this confirms negligible oxidation of DOS_Red_ under anoxic storage or during DOM reisolation. The Dark O_2_ Purge treatment, purged for 8 days with zero-grade air, had similar DOS content (atomic S/C = 9.0 × 10^–3^), DOS speciation (DOS_Red_ = 79%), and SO_4_^2–^ concentration (2.8 μM) as the t = 0 (initial) sample. Differences in DOS_Red_ abundance between these three DOM samples were small and within the accuracy of S K-edge XANES measurements,^16^ and DOM had comparable DOS content (within 6.2%), DOC and SO_4_^2–^ concentrations, and DOM optical properties (Table 1, Figure S7). In summary, DOS_Red_ was completely resistant to dark oxidation, consistent with experiments tracking reaction byproducts^21^ and the observed stability of DOS to redox manipulations.^36^

**Table 1:** Data of Oxidation Experiments Including the Duration, Atomic Sulfur-to-Carbon Ratio (S/C) of Dissolved Organic Matter (DOM), Concentrations and Percentages of DOS Atomic Fractions, Inorganic Sulfate (SO_4_^2–^), and Total Sulfur (S_Tot_)

				DOS Species by S K-edge XANES Spectroscopy^b^,^c^						
Sample	Exp. Duration^a^	[DOC] mgC L^–1^	DOM Atomic S/C^b^	DOS_Red_ μM	DOS_Sulfx_ μM	DOS_SO_2__ μM	DOS_SO_3__ μM	DOS_SO_4__ μM	[SO_4_^2–^]^d^ μM	S_Tot_^e^ μM
*t* = 0 (initial)	—	37.9	9.6 × 10^–3^	24.6	0.2	0.7	3.6	0.9	1.0	31.2
				(82%)	(0.8%)	(2.4%)	(12%)	(2.9%)		(—)
Dark, Anoxic Control	336 h	38.6	9.7 × 10^–3^	24.2	0.5	0.9	4.0	1.8	2.8	34.1
				(77%)	(1.5%)	(2.8%)	(13%)	(5.8%)		(110%)
Dark O_2_ Purge	192 h	37.8	9.0 × 10^–3^	22.4	0.3	0.8	3.7	1.0	2.8	30.9
	30 mL min^–1^			(79%)	(1.2%)	(2.7%)	(13%)	(3.5%)		(99%)
Light-1	1.3 h	35.0	9.4 × 10^–3^	21.0	0.5	0.8	3.8	1.4	2.9	30.3
				(77%)	(1.8%)	(2.9%)	(14%)	(5.1%)		(97%)
Light-2	5.3 h	34.4	8.8 × 10^–3^	18.4	0.5	0.8	3.8	1.6	4.0	29.1
				(73%)	(2.2%)	(3.3%)	(15%)	(6.2%)		(93%)
Light-3	24 h	32.6	7.6 × 10^–3^	14.0	0.7	0.8	3.7	1.6	8.5	29.2
				(68%)	(3.2%)	(3.7%)	(18%)	(7.9%)		(94%)
Light-3 (replicate)	24 h	33.1	7.9 × 10^–3^	15.2	0.8	0.7	3.9	1.3	8.1	30.0
				(69%)	(3.5%)	(3.4%)	(18%)	(6.0%)		(96%)
Light-4	78 h	28.4	6.1 × 10^–3^	8.4	0.5	0.7	3.0	2.0	14.0	28.5
				(58%)	(3.1%)	(4.9%)	(21%)	(14%)		(91%)
Light-5	192 h	27.0	4.9 × 10^–3^	5.5	0.4	0.6	3.1	1.5	21.8	32.9
				(50%)	(3.7%)	(5.6%)	(28%)	(13%)		(106%)

a For light treatment samples the experimental duration is the time in a solar simulator at 500 W m^2–^.

b Measured on DOM extracts.

c Eq 2 used to determine aqueous concentrations. Values in parentheses are atomic fractions (%) of organic sulfur.

d Measured on aqueous solutions prior to DOM extraction.

e Eq 3 used to determine total S concentration.

### Selective Photochemical Oxidation of Reduced DOS

**Figure 2:**
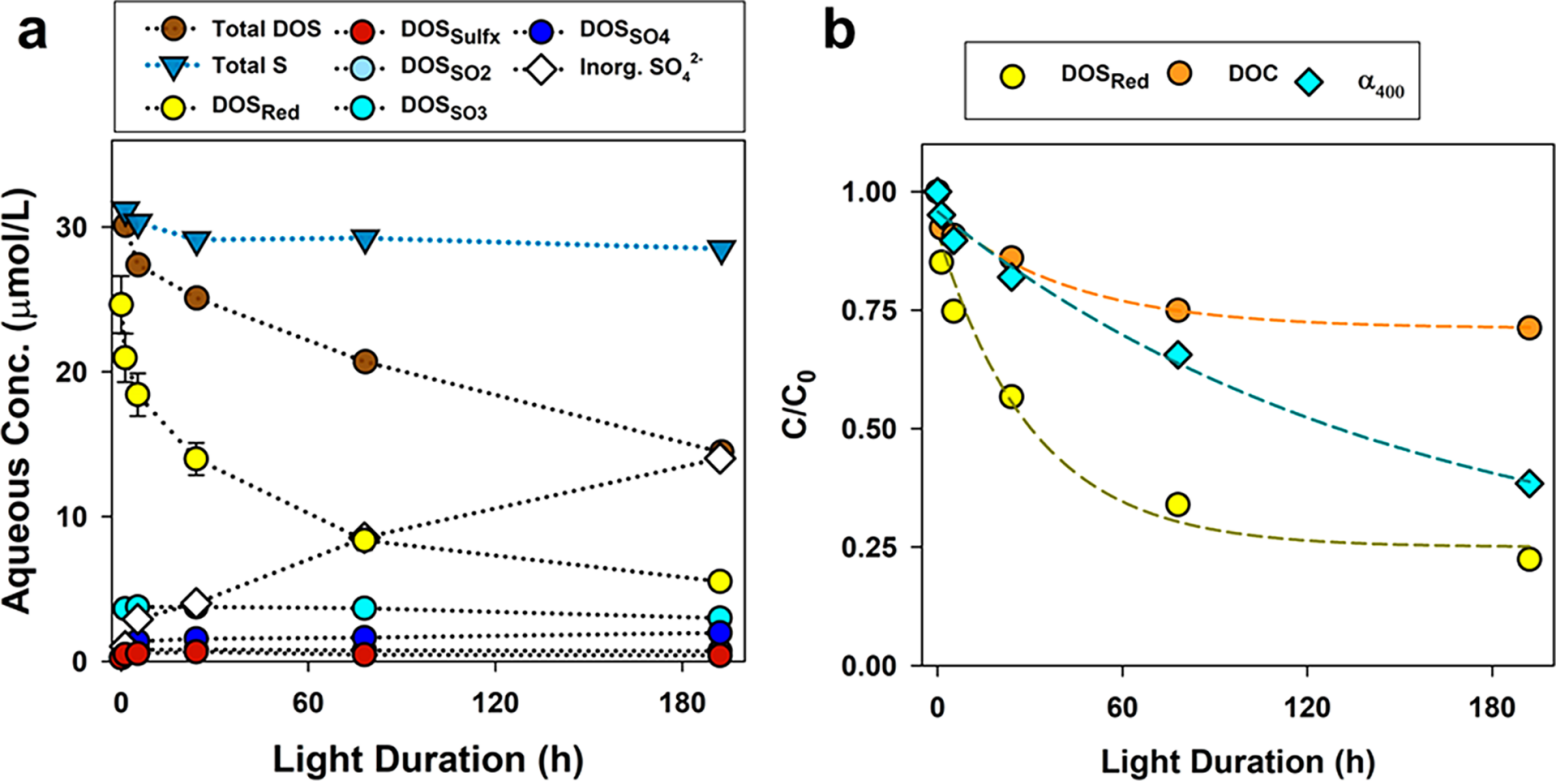
Kinetics results of light treatment (Light 1–5, 1.3–192 h) including the (a) aqueous concentrations of total sulfur (Total S), total organic sulfur (Total DOS), five DOS functionalities quantified by S K-edge XANES spectroscopy (DOS_Red_, DOS_Sulfx_, DOS_SO2_, DOS_SO3_, DOS_SO4_), and inorganic sulfate (SO_4_^2–^). Plot of (b) C/C_0_ showing the rapid rate of DOS_Red_ photodegradation in comparison to DOC photomineralization and DOM photobleaching (shown as α_400_). In plot (a), the decrease in concentrations of DOS_Red_ functionalities with increasing light duration is concurrent with the increase in SO_4_^2–^ concentration; dotted lines are provided to guide the eye, and error bars present accuracies of DOS_Red_. In plot (b), the dashed lines present the exponential fit of the data to guide the eye.

DOM exposure to artificial sunlight yielded systematic and pronounced changes in S K-edge XANES spectra (Figure 1b), atomic fractions of DOS functionalities (Figure S8, Table S4), and DOM S content (Table 1). With increasing cumulative irradiance (light 1–5, 1.3–192 h light exposure), systematic decreases were observed in X-ray absorption at energies of DOS_Red_ functionalities. Fitting results quantified a systematic decrease in DOS_Red_ from 82% of total DOS in the t = 0 (initial) sample to 50% at 192 h of irradiance. An experimental replicate of the Light 3 sample confirmed good reproducibility for each DOS functionality (differences ≤1.7%) (Figure S9, Tables 1 and S4). Simultaneously, a dramatic and systematic decrease was observed in the DOM S content (49% decrease in atomic S/C). Aqueous concentrations of DOS species (eq 2) show dramatic decreases in DOS_Red_ with increasing cumulative irradiance (Figure 2a). In contrast, concentrations of other DOS functionalities (DOS_Sulfx_, DOS_SO2_, DOS_SO3_, and DOS_SO4_) were largely uniform in the light treatment. Decreases in DOS_Red_ concentration accounted for all changes in the DOS_Tot_ concentration (Figure S10), confirming that shifts in S K-edge XANES spectra and decreases in atomic S/C of DOM were exclusively due to the oxidation of DOS_Red_. The decrease in the DOS_Red_ concentration with increasing irradiance was mirrored by a quantitative increase in the SO_4_^2–^ concentration (from 1.0 to 21.8 μM; Figure 2a). Importantly, the DOS_Red_ concentration approached an asymptote, with 37% of the DOS_tot_ being recalcitrant to photochemical oxidation over the experiment, similar to observations made of DOM from diverse sources.^21^ A mass balance analysis of total S in the light experiment (S_Tot_; eq 3) accounted for 93–106% of S_Tot_ at all time points (Table 1), verifying quantitative formation of SO_4_^2–^ concurrent with photochemical oxidation of DOS_Red_.

The light treatment also yielded systematic responses in the DOC concentration and DOM optical indices (Figure S7). Between the t = 0 (initial) and Light 5 sample, the DOC concentration decreased from 37.9 to 27.0 mgC L^–1^, α_254_ and α_400_ values decreased by 60%, and systemic shifts in DOM optical indices were observed including a decrease in DOM SUVA_254_ (from 4.6 to 2.6 L (mgC m)^−1^), increase in *S*_*275–295*_ (from 14.9 to 16.8 x10^–3^ nm^–1^), and decrease in HIX (from 24.2 to 9.4) (Figure S7). These changes in DOC concentration and DOM optical metrics were strictly due to photochemical processes, consistent with previous observations of photomineralization^21^ and photobleaching of DOM chromaphores.^31,33^

Relative rates of photochemical transformations differed drastically between the DOS_Red_ concentration, DOC concentration, and DOM absorption coefficients (e.g., α_400_), as shown in Figure 2b as C/C_0_ versus light exposure. After 5.3 h of irradiance, 25% of the DOS_Red_ was photo-oxidized to SO_4_^2–^ whereas α_400_ and DOC concentration only decreased by 5% and 3%, respectively. After 192 h of irradiance, 78% of the DOS_Red_ was photo-oxidized to SO_4_^2–^. DOS_Red_ oxidation rates could not be adequately modeled using first- or second-order reaction kinetics. The rapid decrease in relative concentration of DOS_Red_ demonstrates the high susceptibility of the majority of DOS_Red_ groups to photochemical oxidation to SO_4_^2–^, notably faster than the photolysis of DOM chromophores and photomineralization of DOC.

The contrasting stability of DOS_Red_ to partial or complete oxidation under dark and light conditions, with little evidence of photochemical oxidation or accumulation of intermediate DOS species (e.g., DOS_SO2_, DOS_SO3_, and DOS_SO4_), could be explained by specific DOS_Red_ chemistry or mechanisms of oxidative protection. At the start of the experiment, DOS_Red_ was likely present as a mixture of thiol and thioether groups, which both could originate from sulfurization reactions^3,9^ or biomolecules (e.g., cysteine and methionine) and are known to undergo photochemical oxidation to SO_4_^2–^.^21^ Previous measurements of DOM from this wetland confirmed that 98% of molecules that made up DOS_Red_ had one S atom (e.g., CHOS_1_, CHON_1–2_S_1_),^1^ discounting the prominence of disulfide moieties. Further, thiols are confirmed in DOM from diverse aquatic environments including sulfidic wetlands and lakes, based on measured binding configuration^37^ and strength of DOM-mercury complexes^19^ but account for a fraction of DOS_Red_ based on a mercury-titration study.^34^ Yet, model thiols undergo rapid dark oxidation,^17,18^ which contrasts with the dark stability of DOS_Red_ observed here. Perhaps DOM_Red_ as thiols are protected from dark oxidation by O_2_ in hydrophobic DOM pockets^19^ but when exposed to sunlight rapidly oxidize due to high concentrations of photoreactive species (e.g., triplet excited state DOM (^3^CDOM*)).^38^ This would explain the observed susceptibility of DOS_Red_ to sunlight. Although the distribution of thiol and thioethers that make up DOS_Red_ could not be resolved here, the observed complex kinetics of DOS_Red_ photochemical oxidation and previous mechanistic studies support that a combination of direct photolysis of chromophoric DOS_Red_^21^ and indirect photolysis via triplet excited state DOM (^3^CDOM*)^22^ explains the photochemical oxidation of thiols and thioether groups to SO_4_^2–^.

The finding of selective DOS_Red_ photochemical oxidation to SO_4_^2–^ agrees with irradiance studies of low-molecular-weight thiols and thioethers^21^ and DOM, quantified by either the production^21^ of SO_4_^2–^ or loss of S-containing molecules.^23−25^ Selective photochemical oxidation of DOS_Red_ to SO_4_^2–^ was inferred by Ossola et al. (2019),^21^ as this pathway was greatest in DOM collected from sulfidic environments. Further, a separate analysis presented in Figure S11 shows that the photochemical oxidation of DOS_Red_ to SO_4_^2–^ measured of IHSS samples^21^ is greatest in DOM with elevated %DOS_Red_, the latter measured by Manceau and Nagy (2012).^16^ Photochemical oxidation of DOS_Red_ to SO_4_^2–^ may occur through organic (DOS_SO2_, DOS_SO3_) or inorganic intermediates (SO_2_, SO_3_^2–^),^21^ which may not have accumulated in experimental solutions or may have been at a low concentration. It is unclear why a fraction of DOS_Red_ was photorecalcitrant (Figure 2a, Table 1), but this observation is consistent with previous laboratory studies.^21,25^ Metals have been observed to prevent^18^ and promote^39^ oxidation of model reduced S compounds, but additional investigations are required with DOS. Similarly, oxidized organic S functionalities (e.g., DOS_SO3_) did not change in concentration due to irradiance. Perhaps DOS_SO3_ groups are primarily in nonchromophoric DOM molecules, as supported by photochemical oxidation experiments of model compounds,^21^ or that their relative low concentration obscured detection. Results from this study provide a critical atomic-level validation of mechanisms of the selective and rapid photochemical oxidation of DOS_Red_ to SO_4_^2–^.

### Implications of Findings in Biogeochemical Cycles

The selective photochemical degradation of DOS_Red_ to SO_4_^2–^ observed in DOM from sulfidic pore waters is likely an important phenomenon in fresh and marine surface waters, and is likely a result of formation^1,3,9^ and stabilization of DOS_Red_ in DOM moieties that are highly susceptible to photochemical oxidation. The oxidation of DOS_Red_ helps explain why methylmercury (with Hg in a divalent oxidation state), exclusively bound to DOM thiols in freshwaters, is photoreduced to gaseous elemental Hg rather than photodegraded to divalent inorganic Hg.^7,8^ DOS_Red_ may be a precursor to minor volatile organic S species not measured here (e.g., COS, CS_2_, DMS),^4^ whereas oxidized DOS functionalities (e.g., DOS_SO2_, DOS_SO3_) could be precursors of methanesulfonic acid and methanesulfinic acid;^21^ both require future investigation. Yet, the high relative abundance of DOS_Red_ in photic freshwater systems^1,16,19^ remains an enigma. The photostability of DOS as sulfonate (DOS_SO3_) here contrasts conclusions drawn of marine and wetland DOS speciation and photolability using a molecular derivatization analysis,^25,40^ highlighting the need for coupled atomic- and molecular-level measurements to unravel DOS complexities in natural waters. This is of particular importance in sulfur-enriched riverine and coastal environments receiving agricultural runoff^41^ and wastewater effluent^10^ and marine waters where DOS (de)sulfurization influences S cycling^2^ and carbon diagenesis.^14^ Future studies are needed to constrain DOS_Red_ speciation and quantify mechanisms and kinetics of DOS_Red_ photochemical oxidation across a variety of aquatic environments.

## Supporting Information Available

Description of DOM extraction, laboratory experiments, chemical analyses, and S-XANES spectra acquisition and processing (PDF)
All S-XANES spectra (XLSX)
