# Supplementary material for: Selective Photochemical Oxidation of Reduced Dissolved Organic Sulfur to Inorganic Sulfate
Source: Environ Sci Technol Lett. 2023 May 3;10(6):499–505. doi: 10.1021/acs.estlett.3c00210 (PMC10275504; doi:10.1021/acs.estlett.3c00210)
Supplement: Supplementary file 1 — ez3c00210_si_001.pdf [file ez3c00210_si_001.pdf]

Supporting Information

to

Selective Photochemical Oxidation of Reduced Dissolved Organic Sulfur to Inorganic Sulfate

Brett A. Poulin[a*]

[a]Department of Environmental Toxicology, University of California Davis, Davis, CA 95616,

USA

* Corresponding author. Tel: +1 530 754 2454. *Email address:* bapoulin@ucdavis.edu

Supporting information includes 11 Figures, 4 Tables, and 22 pages

Table of Contents

Section S1. Description of Experimental Steps and Analyses 2

a. DOM Sample Collection and Extraction 2

b. Laboratory Oxidation Experiments 3

c. DOC Concentration and DOM Optical Analyses 4

d. Inorganic Sulfur Analyses 5

e. Sulfur XANES Spectra Acquisition and Processing 5

Section S2. Evaluation of the DOM Solid Phase Extraction 6

Section S3. Supplemental Figures & Tables (Figures S1-S11, Table S1-S4) 8

References 22

Section S1. Description of Experimental Steps and Analyses

a. DOM Sample Collection and Extraction

Figure S1 provides a flow diagram of the steps taken in the (1) collection of the DOM sample from the field for laboratory experimentation, (2) laboratory isolation of DOM, and (3) laboratory oxidation experiments. A pore water sample was collected from a highly sulfidic location in Water Conservation Area 2A of the Florida Everglades (site WCA 2A-O; 26.42506°N, -80.47601°W). DOM from this location is enriched in reduced sulfur species due to sulfurization reactions with inorganic sulfide, as previously shown.[1] Pore water (30 L) was collected 10 cm below the sediment-water interface at a rate of 100 mL min^{-1} using a Teflon sipper connected to Teflon tubing and a peristaltic pump. Pore water temperature, conductivity (Orion four-cell conductivity electrode), pH (Orion ROSS Ultra™ electrode), dissolved oxygen (DO) concentration (Orion RDO optical probe), and oxidation-reduction potential (ORP; Orion ORP Triode electrode) were measured using a flow-through cell (Geotech; 40 mL dead volume) and multi-parameter meters (Orion Star™ A329, Beckman Coulter pHi 410). Electrodes were calibrated using manufacturer specifications prior to field deployment. All measurements collected during the sampling are reported in an associated data release.[2] The sipper was repositioned laterally by approximately 0.5 m every 10 min to not deplete sediment pore waters. The pore water was field filtered (0.45 µm polysulfone capsule filters; Geotech) directly into 2 L polyethylene terephthalate bottles with no head space. The sample was stored at 4 °C and shipped on ice to the USGS (Boulder, Colorado) for DOM isolation.

In the laboratory, care was taken to minimize exposure of the pore water sample to atmospheric oxygen during storage and DOM isolation. First, residual inorganic sulfide (H_2S/HS^-; pK_a = 7.0) was removed (to <0.3 µM) from the sample by adjusting to pH 4.0 with trace-metal grade HCl and purging with helium for 1 h. Sulfide removal was confirmed by preserving a subsample with 50% volume/volume sulfide anti-oxidant buffer and measuring S^{2-} by ion-elective electrode (Orion silver/sulfide; calibrated using 0.3 – 310 µM calibration standards). Next, the hydrophobic organic acid (HPOA) fraction of DOM was isolated on XAD-8 resin with precautions taken to minimize possible oxidation of organic sulfur. All solutions used during the SPE were degassed with helium for 1 hour prior to use. During isolation, the sample was back-eluted with deaerated 0.1 M NaOH directly through proton-saturated cation exchange resin. Experimental results provided in Section S2 of the Supporting Information demonstrate that the isolation of DOM by XAD-8 resin using deaerated solutions did not result in measurable oxidation of organic sulfur compared to XAD-8 isolation using solutions at equilibrium with the ambient atmosphere

or an alternative SPE procedure that uses a methanol elution (**Figure S2**).[3] The HPOA sample accounted for 54% of the dissolved organic carbon (DOC) in the whole water sample and was stored for 21 days (pH 3.5, under nitrogen, 4 °C) until use in laboratory oxidation experiments.

b. Laboratory Oxidation Experiments

First, the purified DOM sample was diluted with deaerated high-purity water (≥18 MΩ cm; Barnstead GenPro UV) to a DOC concentration of 37.9 mg L^{-1} and adjusted to pH 7 with deaerated 0.1 M NaOH solution (**Figure S1**). Differences in ionic strength are not expected to influence rates of DOS oxidation,[4] and therefore no other inorganic salts were added to the starting DOM solution. A sub-sample (1 L volume) was immediately preserved for analysis (termed t = 0 (initial)) to characterize the initial experimental conditions. Inorganic sulfate was quantified at 1.0 µM and accounted for approximately 3% of total sulfur at the start of the experiment. The following four treatments were performed in 2 L quartz round bottom flasks filled with 1.0 L of DOM solution.

1. A dark anoxic control treatment (*n* = 1, termed Dark, Anoxic Control) was stored in the dark (vessel covered in aluminum foil), under nitrogen at 22±2 °C for 14 d to identify changes in sulfur speciation during dark storage or DOM re-isolation on XAD-8 resin.

2. A dark O_2 purge treatment (*n* = 1, termed Dark, O_2 Purge) was purged in the dark (vessel covered in aluminum foil) with zero-grade air (20.5% oxygen, 79.5% nitrogen) at 30 mL min^{-1} at 22±2 °C for 192 h. This treatment quantified the susceptibility of reduced organic sulfur species in DOM to oxidation by molecular oxygen, and the purge duration was equivalent to the duration of the longest light treatment (Light 5).

3. Light treatment (*n* = 5, termed Light 1-5) were performed in a temperature-controlled solar simulator at 30 °C (Suntest XLS; calibrated prior to use). The Light 3 treatment was conducted in duplicate to assess experimental reproducibility. The irradiance was 500 W m^{-2} (300 – 800 nm irradiance range) at the sample shelf of the solar simulator (spectrum provided in **Figure S3**). The solution surface was 10.5 cm above the sample shelf. The photon absorption rate could not be used to determine the photo-irradiance due to changes in DOM absorption[4] over long light experiment. Immediately prior to irradiation, experimental solutions were purged with zero-grade air for 15 minutes to ensure dissolved oxygen saturation (75 mL min^{-1}). The dissolved oxygen concentration was measured at 7.1 mg L^{-1} (98% saturation; Orion RDO optical probe) prior to the start of the experiment. Independent vessels were

irradiated for the following times: 1.3 h, 5.3 h, 24 h, 78 h, and 192 h. For Light treatments of 24, 78, and 192 h experimental duration, solutions were purged with zero-grade air every 12 hours for 15 minutes (75 mL min^{-1}) to ensure oxygen saturation. The rationale was to maintain consistent experimental conditions, due to previous observations of dissolved oxygen depletion during DOM photolysis.[4]

At the end of oxidation experiments, solutions were sampled for aqueous measurement of DOC concentration, DOM absorption and fluorescence spectra, inorganic sulfate (SO_4^{2-}), and thiosulfate ($S_2O_3^{2-}$). Aliquots for DOC concentration, DOM absorption and fluorescence spectra, and inorganic sulfate were stored at 4 °C in pre-baked (450 °C for 4.5 h) amber borosilicate glass vessels with Teflon®-lined caps. Aliquots for thiosulfate measurement were stored in high-density polyethylene bottles preserved with zinc acetate (1% volume/volume Zn acetate) and NaOH (1% volume/volume 1 M NaOH). Next, solutions were deaerated by purging with nitrogen for 1 hour, stored under nitrogen, and DOM was re-isolated by SPE on XAD-8 resin taking precautions to minimize oxidation (as described above). The average recovery of DOC during the re-isolation was 85%; there was no evidence of DOM fractionation based on spectroscopic measurements of the DOM before and after re-isolation on XAD-8 resin. DOM extracts were lyophilized and stored under nitrogen. Inorganic SO_4^{2-} was confirmed to be below the detection limit (<0.50 µM) in DOM extracts (as describe below in Section S1d), confirming that S XANES spectra strictly measured DOS species.

c. DOC Concentration and DOM Optical Analyses

DOC concentration was determined by persulfate oxidation (OI Analytical, model 700).[5] UV-vis absorption spectra were measured from 190-800 nm using a spectrophotometer (Agilent Technologies, model 8453) and a 1 cm quartz cuvette. Sample UV-vis spectra were measured with respect to a blank spectrum containing high-purity water (≥18 MΩ cm; Barnstead GenPro UV). Decadic absorbance coefficients at 254 nm (α_{254})and 400 nm (α_{400}) were quantified using Equation S2, where α_λ is the decadic absorption coefficient (cm^{-1}), A_λ is the absorbance, and l is the path length (cm).

$$\alpha_\lambda = \frac{A_\lambda}{l} \qquad \text{(S2)}$$

The specific ultraviolet absorbance at 254 nm ($SUVA_{254}$), a proxy for DOM aromaticity,[6] was calculated by dividing the α_{254} by DOC concentration and are reported in units of L (mgC $m)^{-1}$. The spectral slope between 275-295 nm ($S_{275\text{-}295}$) was calculated by fitting the exponential Equation S3 to the Napierian absorption spectra between wavelengths of 275 – 295 nm:

$$\alpha_{g(\lambda)} = \alpha_{g(\lambda_{ref})} e^{-S(\lambda - \lambda_{ref})} \quad \text{(S3)}$$

where $\alpha_{g(\lambda)}$ is the absorption coefficient at the specified wavelength, $\alpha_{g(\lambda_{ref})}$ is the absorption coefficient at the reference wavelength, and S is the slope fitting parameter.[7] Excitation emission matrix fluorescence spectra were collected by scanning samples between excitation 240 – 450 nm at 5 nm intervals and emission 300 – 600 nm at 2 nm intervals using a Jobin Yvon Horiba Fluoromax 3 fluorometer. Fluorescence spectra, reported in Raman units (RU), were corrected for UV-Vis absorption, DI blank, and Raman scatter. The humification index (HIX) is defined as the area under the emission spectrum from 435 to 480 nanometers divided by the summation of areas under the emission spectrum at 300 to 345 nanometers and 435 to 480 nanometers, at an excitation of 254 nanometers.

d. Inorganic Sulfur Analyses

Inorganic sulfate (SO_4^{2-}) and thiosulfate ($S_2O_3^{2-}$) were quantified by ion chromatography (IC; Dionex IonPac® AS14A column).[8] Sulfate was quantified using a six point calibration curve (0.5-200 µM) and two custom quality control solutions (Inorganic Ventures). The detection limits for inorganic sulfate and thiosulfate were 0.50 µM and 0.45 µM, respectively, based on the concentration of the lowest calibration standard.

e. Sulfur XANES Spectra Acquisition and Processing

The sulfur and carbon contents of freeze-dried DOM samples, used to quantify the atomic sulfur-to-carbon ratio (atomic S/C), were determined by Huffman Hazen Laboratories (Golden, CO). Sulfur K-edge XANES spectra were collected on DOM samples (n = 12) on beamline 9-BM-B of the Advanced Photon Source (APS) (Argonne National Laboratory) as detailed in Poulin et al. (2017).[1] DOM samples were pressed as 5 mm pellets under ambient atmosphere, stored under nitrogen, and mounted immediately before analysis on sulfur-free carbon tape. A comparison of methods in DOM sample preparation (dispersed DOM powder versus pressed pellet) verified that pressing DOM samples as pellets did not influence the sulfur K-edge XANES spectra (**Figure S4**). Eight scans per sample were collected (2440-2538 eV), moving the sample by 0.5 mm to examine previously unexposed material. Spectra were collected in fluorescence yield mode under a He atmosphere using a Vortex ME4 detector. Using Athena,[9] scans were averaged, normalized to the absorption from 2515.0-2542.5 eV, and fit by Gaussian curve fitting (GCF).[10] Atomic fractions of sulfur functionalities ($f_{\mathrm{DOS_X}}$), including exocyclic reduced

(DOS_{Exo}), heterocyclic reduced (DOS_{Hetero}), sulfoxide (DOS_{sulx}), sulfone (DOS_{SO2}), sulfonate (DOS_{SO3}), and organosulfate (DOS_{SO4}), were calculated with a precision estimated at ≤ 1.6% by measurement of DOM samples from the IHSS.[10] Accuracies of atomic fractions of reduced (DOS_{Exo}, DOS_{Hetero}) and oxidized sulfur functionalities (DOS_{SO2}, DOS_{SO3} , DOS_{SO4}) are estimated at 8% and 4%, respectively.[10] **Figure S5** presents S XANES spectra of 4 model reduced S compounds with differing S functionalities, including cysteine (e.g., thiol), methionine (e.g., thioether), cystine (e.g., disulfide), and dibenzo thiophene (e.g., thiophene). Due to challenges in resolving the exact species of DOS_{Red}, a product of the differences in X-ray absorption peak locations and features (e.g., doublets and shoulders), this study presents the total DOS_{Red}, defined as the summation of DOS_{Exo} and DOS_{Hetero}.

Section S2. Evaluation of the DOM Solid Phase Extraction

Three solid phase extraction (SPE) procedures were compared on a single water sample to assess if changes in organic sulfur speciation may occur as a result of the DOM isolation, specifically during the base-elution step during XAD-8 resin isolation.

1. **XAD-8 Isolation Under Ambient Atmosphere**. The SPE of the HPOA fraction of DOM was carried out on XAD-8 resin (acrylic ester) using solutions (ultrahigh purity water, ≥18 MΩ cm; 0.1 M NaOH, 0.1 M HCl, trace-metal grade) at equilibrium with ambient atmosphere.[5] In brief, the whole water sample was adjusted to pH 2 with trace-metal grade HCl, degassed with helium (15 minutes) per the method,[5] and loaded onto a 300 mL pre-cleaned XAD-8 column at 65 mL min^{-1}. Chloride was removed from the HPOA fraction retained on the XAD-8 column by rinsing with high-purity water at approximately 40 mL min^{-1} until the conductivity of the effluent was < 700 µS cm^{-1}. The sample was back-eluted off the resin with 0.1 M NaOH at 40 mL min^{-1} through a proton-saturated cation exchange resin (CER; Bio-Rad Laboratories) to remove sodium ions. The aqueous HPOA sample was purged with nitrogen and stored under nitrogen prior to lyophilization; this was done to isolate the potential for DOM S oxidation during the SPE step. After lyophilization, the solid DOM sample was stored under nitrogen until further analysis.

2. **XAD-8 with Deaerated Solutions**. SPE of the HPOA fraction of DOM was carried out as described above with the following additional steps taken:
   a. The sample was loaded onto a 300 mL pre-cleaned XAD-8 column containing 0.1 M HCl prepared with deaerated water.

b. Chloride was removed from the HPOA fraction retained on the XAD-8 column by rinsing with deaerated high-purity water.
c. The sample was back-eluted off the XAD-8 resin with 0.1 M NaOH prepared with deaerated water through proton-saturated cation exchange resin pre-flushed with deaerated high-purity water.

3. **PPL Extraction with Deaerated Solutions.** SPE of DOM was carried out on PPL resin following the method of Dittmar et al. (2008).[3] In brief, the sample was adjusted to pH 2 with trace-metal grade HCl, degassed with helium (15 minutes), and loaded onto a PPL column (styrene-divinylbenzene polymer, Agilent). The sample was rinsed with 0.01 M HCl prepared with deaerated water, dried with nitrogen, eluted with deaerated methanol, and dried under a nitrogen gas stream. The solid DOM sample was stored under nitrogen until further analysis.

The DOM samples were analyzed for elemental composition (C, H, S content) and S speciation by S-k-edge XANES spectroscopy (**Tables S1-S2**, **Figure S2**). Negligible differences were observed between the spectra (**Figure S2**) and distribution of sulfur functionalities (**Table S1**) between samples isolated on XAD-8 resin using solutions under ambient atmosphere versus deaerated. The DOM sample isolated using deaerated solutions had a modest 6% higher sulfur content relative to carbon (atomic S/C), but differences in the atomic fractions of sulfur functionalities were within the precision of spectral fits estimated previously to be ≤ 1.6% for a given functionality.[1] Compared to the sample isolated on XAD-8 resin with deaerated solutions (atomic S/C = 10.1 x 10^{-3}, $\mathrm{DOS_{SO_3}}$ = 15%), the sample isolated on PPL resin had a slightly (1) lower sulfur content (atomic S/C = 8.0 x 10^{-3}) and higher percentage of sulfonate functionality ($\mathrm{DOS_{SO_3}}$ = 17%). This observations is consistent with previous studies[4,11] that observed minor differences in the sulfur content of DOM between whole waters and PPL extracts. Yet, differences in DOS functionality between the DOM isolated on XAD-8 versus PPL resin were relatively minor and within the accuracy of atomic fractions of reduced (8%) and oxidized sulfur functionality (4%) estimated by independent methods of spectral fitting.[10] It was previously proposed that PPL resin may not extract DOM with reduced DOS functionalities,[12] yet this is entirely inconsistent with our experiment confirmed ~74% of the DOS was $\mathrm{DOS_{Red}}$. If the isolation of DOM on XAD-8 resin was inducing oxidation of reduced sulfur species, the PPL extract would have a higher percentage of reduced sulfur functionalities, which is inconsistent with the data of this experiment. Therefore, this experiment validates the isolation of DOM on XAD-8 resin with deaerated solutions for the laboratory experiments focused on DOM S oxidation.

Section S3. Supplemental Figures & Tables

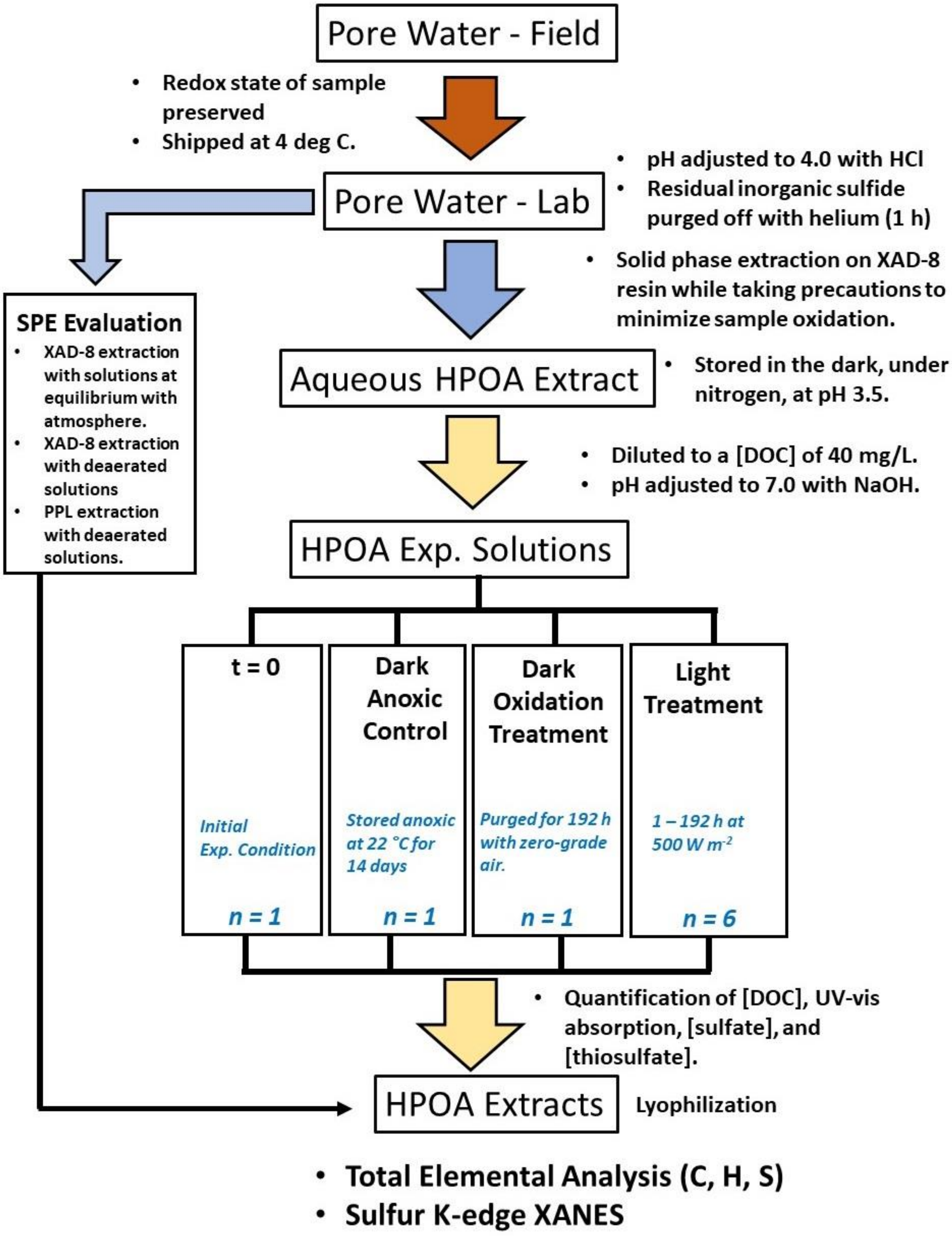

Figure S1. A flow diagram with steps taken for DOM field collection, isolation, and laboratory experimentation.

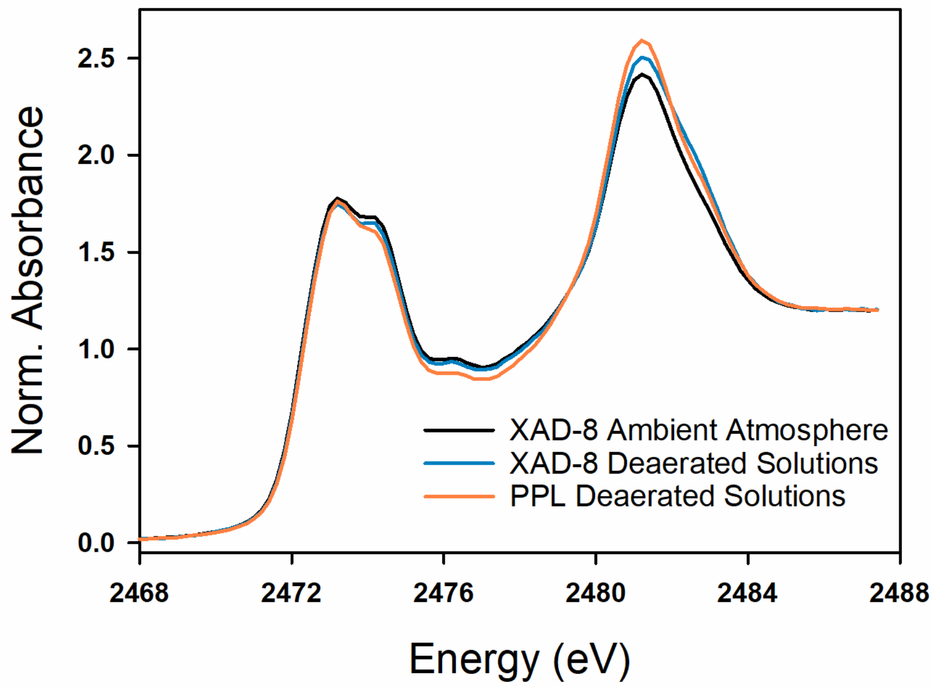

Figure S2. Comparison of S K-edge XANES spectra of DOM isolated using three different solid-phase extraction (SPE) protocols including XAD-8 Ambient Atmosphere (IS17-0004FL HPOA; black line), XAD-8 Deaerated Solutions (IS17-0005FL HPOA; blue line), and PPL Deaerated Solutions (WCA 2A-O PPL; orange line). Minor differences observed between spectra of DOM samples isolated on XAD-8 resin are within the precision of the S XANES fitting. The spectra of DOM isolated on PPL resin (orange line) exhibited modestly greater relative absorption of oxidized sulfur functionalities compared to DOM samples isolated on XAD-8 resin. Elemental data, parameter values for Gaussian curve fitting (GCF), and sulfur atomic fractions are provided in **Tables S1-S2**.

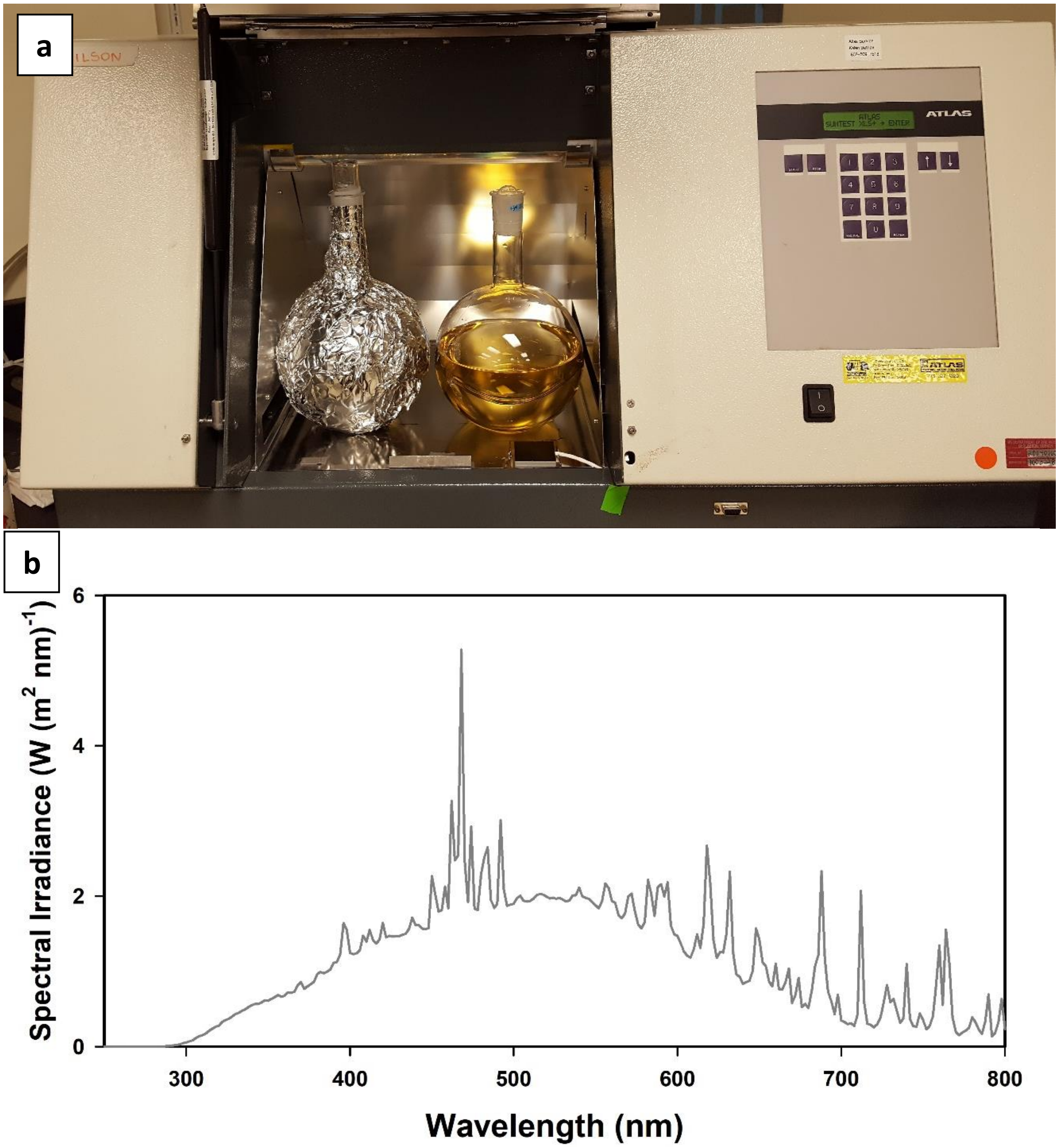

Figure S3. (a) Photo of 2 L quartz flasks for the Dark Control and Light treatments in a Suntest XLS) and (b) the irradiance spectrum of the Suntest XLS solar simulator.

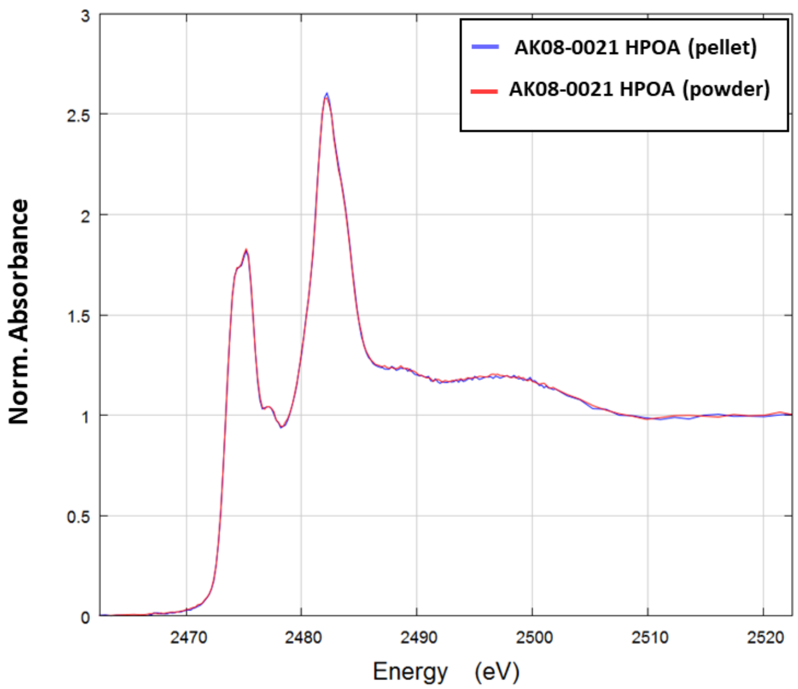

Figure S4. A comparison of sulfur K-edge spectra collected of a DOM sample (AK08-0021 HPOA) prepared as a pressed pellet (blue spectrum) versus a dispersed powder (red spectrum).

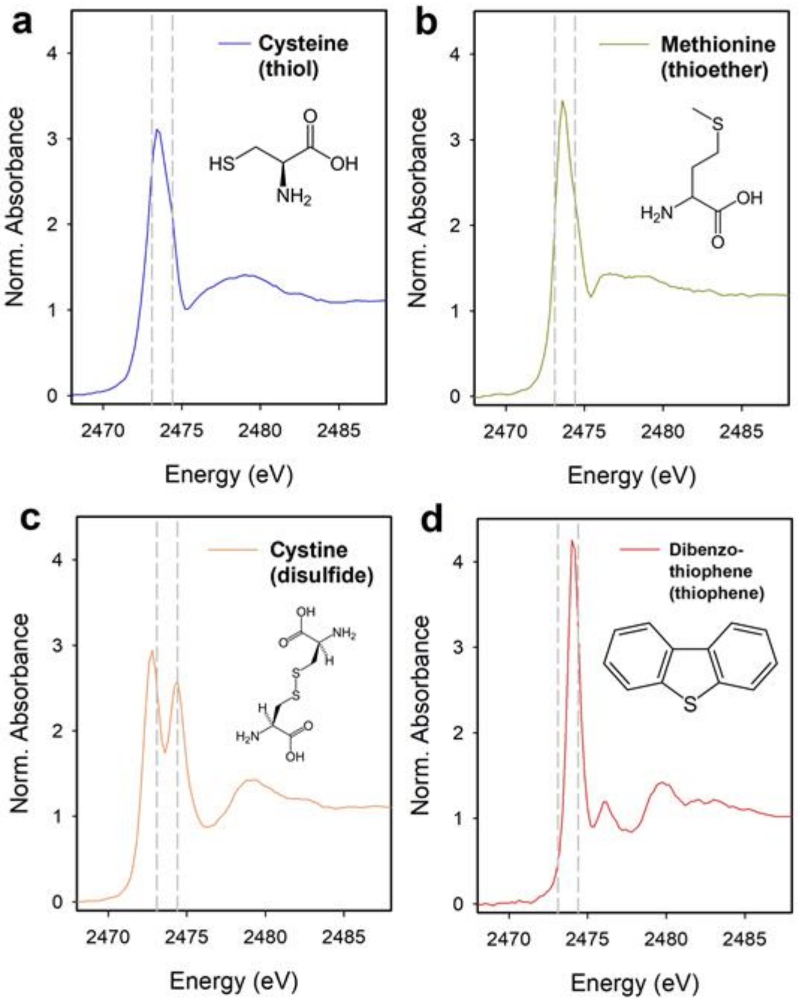

Figure S5. A comparison of sulfur K-edge spectra of model reduced organic sulfur species, including (a) cysteine (thiol), (b) methionine (thioether), (c) cystine (disulfide), and (d) dibenzothiopene (thiophene). Gray dashed vertical lines identify nominal energies of DOS_{Red} (E_0 = 2473.1 and 2474.4 eV for exocyclic and heterocyclic reduced DOS). Spectra are from Manceau and Nagy (2012).[10]

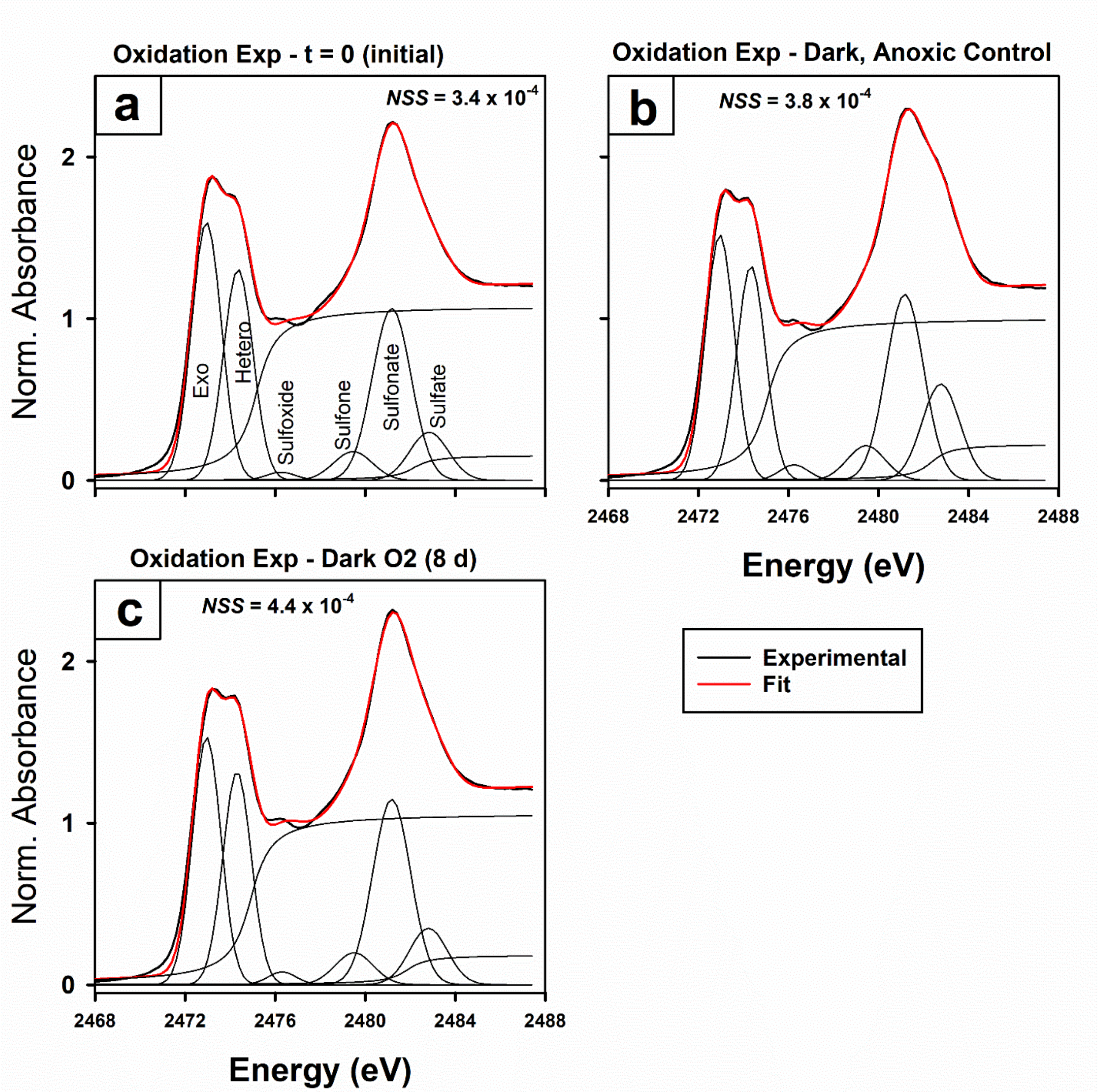

Figure S6. Decomposition of sulfur K-edge XANES spectra of experimental hydrophobic organic acid (HPOA) DOM samples including (**a**) t = 0 (initial), (**b**) dark, anoxic control, and (**c**) dark O_2 (192 h) with two arctangent and six Gaussian functions. The normalized sum-squared residual (*NSS*) is the normalized difference between experimental (black lines) and fit spectra (red lines). Plots **a-c** show negligible changes in the relative abundance of sulfur functionalities between the initial experimental conditions (**a**), control sample (**b**), and when purged with zero-grade air for 192 h (**c**). Sulfur functionalities corresponding to the six Gaussian functions are labeled in plot **a**. The parameter values and atomic fractions of sulfur functionalities of fit spectra are provided in **Table S3**.

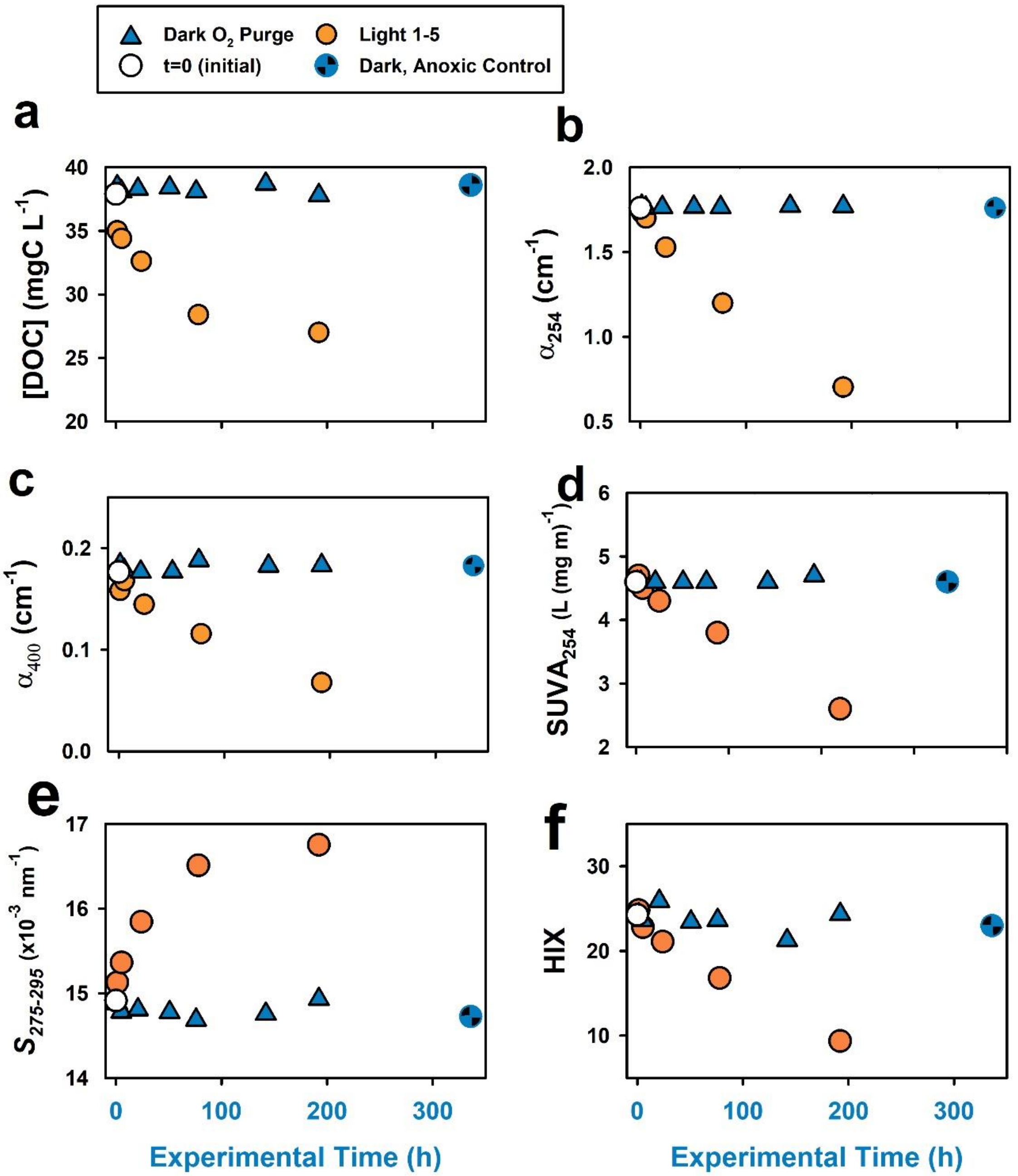

Figure S7. Plots comparing the kinetics of DOC concentration, decadic absorption coefficients at 254 nm (α_{254}) and 400 nm (α_{400}), and DOM UV-vis and fluorescence indices ($SUVA_{254}$, $S_{275-295}$, HIX) of oxidation treatments (Light 1-5, Dark O_2 Purge) to the initial sample (t = 0 (initial)) and Dark Anoxic Control. Plot (**a**) presents [DOC] (in units of mgC L^{-1}) versus time, (**b-c**) present the α_{254} and α_{400} versus time, and (**d-f**) present changes in DOM optical indices ($SUVA_{254}$, $S_{275-295}$, HIX) versus time. In all plots, the experimental time of Light treatments is the duration of irradiance.

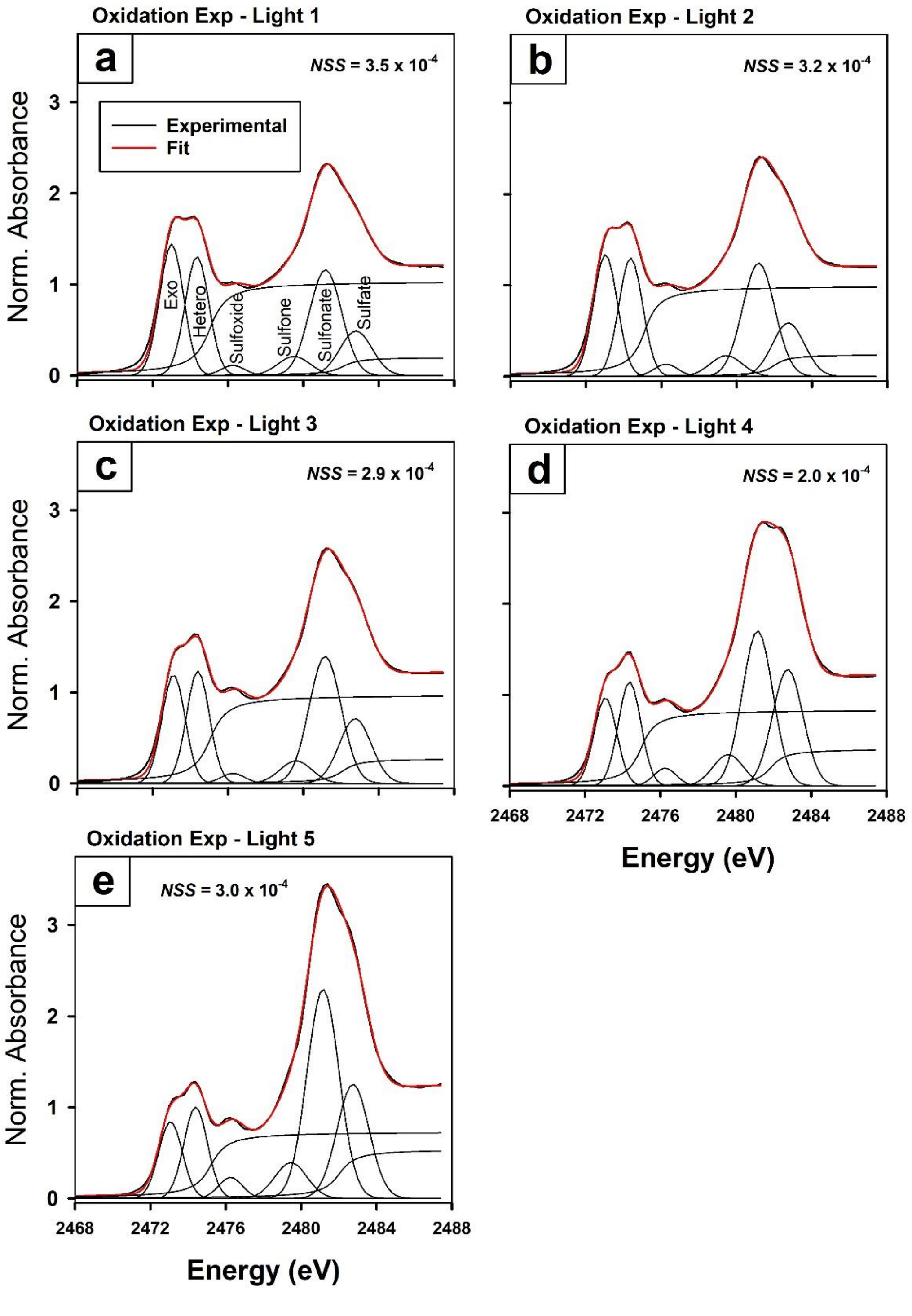

Figure S8. Decomposition of sulfur K-edge XANES spectra of experimental hydrophobic organic acid (HPOA) DOM samples including (**a-e**) Light treatment 1-5 (1.3 - 192 hours) with two arctangent and six Gaussian functions. The normalized sum-squared residual (*NSS*) is the normalized difference between experimental (black lines) and fit spectra (red lines). Plots **a-e** show a systematic decrease in the relative abundance of exocyclic reduced sulfur ($\mathrm{DOS_{Exo}}$) and increase in sulfonate ($\mathrm{DOS_{SO_3}}$) with increased irradiance time. Sulfur functionalities corresponding to the six Gaussian functions are labeled in plot **a**. The parameter values and atomic fractions of sulfur functionalities of fit spectra are provided in **Table S4**.

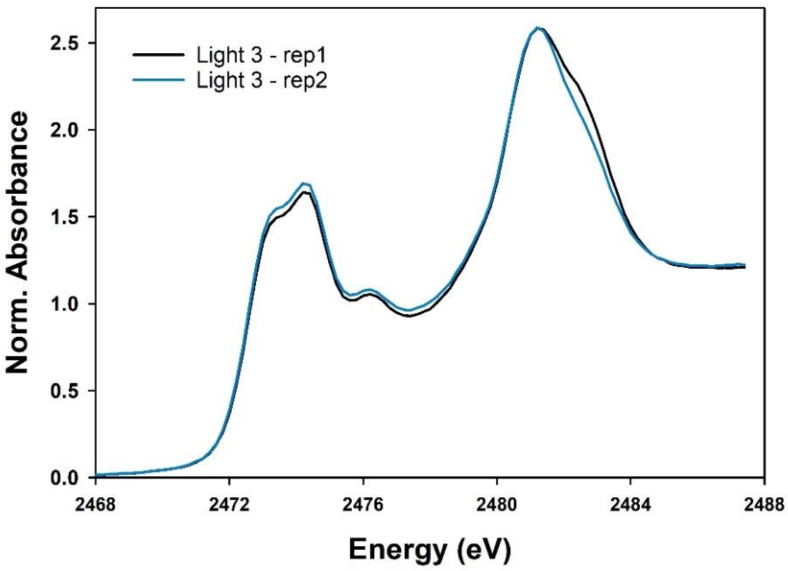

Figure S9. Comparison of sulfur K-edge XANES spectra of replicate Light 3 samples. The fitting results (**Table S4**) show that the distribution of DOS functionalities was within 1.7% between the experimental replicates.

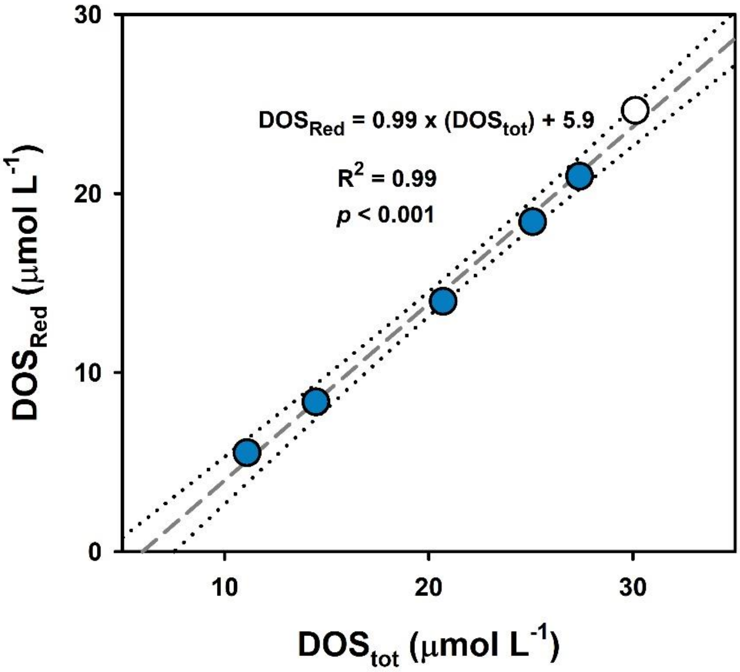

Figure S10. The linear correlation between the concentration of dissolved reduced organic sulfur (DOS_{Red}; Equation 1 in the main text) and total organic sulfur (DOS_{tot}) of the t=0 (initial) sample (white symbol) and Light 1-5 samples (blue symbols). The dashed gray line is the linear fit of the data and the dotted black lines correspond to the 95% confidence intervals of the fit. The slope of the regression is 0.99, indicating that photochemical desulfurization solely resulted in the oxidation and loss of DOS_{Red} from DOM.

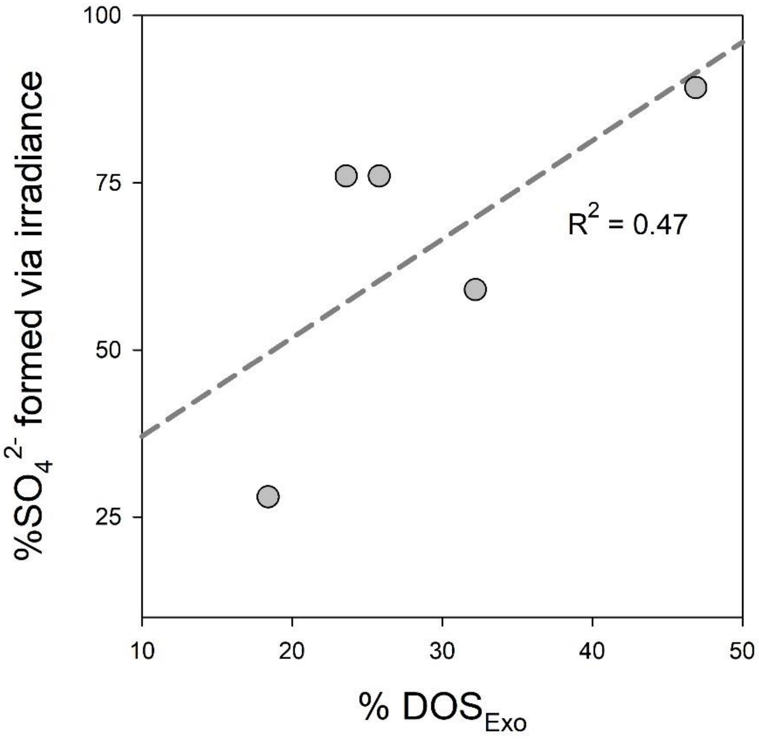

Figure S11. Scatter plot and regression between the percentage of highly-reduced dissolved organic sulfur (%DOS_{Exo}; from Manceau & Nagy (2012)[10]) and the percentage of SO_4^{2-} formed from photo-irradiance experiments of IHSS DOM samples (from Ossola et al. (2019)[4]). IHSS samples include Suwannee River Humic Acid (2S101H), Suwannee River Fulvic Acid (2S101F), Nordic Aquatic Humic Acid (1R105H), Pony Lake Fulvic Acid (1R109F), and Elliot Soil Humic Acid (1S102H). The dashed gray line is the linear fit of the data.

Table S1. Parameter values for Gaussian curve fitting (GCF) and sulfur atomic fractions (%) of measured spectra of DOM isolated using three separate solid-phase extraction (SPE) procedures. Measured spectra of these three samples are presented in **Figure S2**.

**Function**	**S Functionality**	**Energy (eV)**[a]	**Width**[a]	**Calib. Area**[b]	**DOS Atomic Fraction ($f_{\mathrm{DOS_X}}$)**[c] **(%)**	**Function**	**S Functionality**	**Energy (eV)**[a]	**Width**[a]	**Calib. Area**[b]	**DOS Atomic Fraction ($f_{\mathrm{DOS_X}}$)**[c] **(%)**
**XAD-8 Ambient Atmosphere (*IS17-0004FL HPOA*)**						**XAD-8 Deaerated Solutions (*IS17-0005FL HPOA*)**					
Arc 1		2475.1	**0.61**			Arc 1		2475.0	**0.62**		
Arc 2		2482.2	**0.61**			Arc 2		2482.1	**0.62**		
G1	Exocyclic S	2472.9	**0.65**	2.30	50	G1	Exocyclic S	2472.9	**0.64**	2.19	48
G2	Heterocyclic S	**2474.4**	**0.65**	1.29	28	G2	Heterocyclic S	**2474.3**	**0.64**	1.24	27
G3	Sulfoxide	**2476.3**	**0.65**	0.05	1.0	G3	Sulfoxide	**2476.3**	**0.64**	0.04	0.9
G4	Sulfone	**2479.5**	**0.85**	0.13	2.7	G4	Sulfone	**2479.5**	**0.84**	0.14	3.0
G5	Sulfonate	**2481.2**	**0.85**	0.68	15	G5	Sulfonate	**2481.2**	**0.84**	0.71	16
G6	Organosulfate	**2482.8**	**0.85**	0.19	4.1	G6	Organosulfate	**2482.8**	**0.84**	0.24	5.3
NSS[d] = 3.9×10^{-4}						NSS[d] = 3.7×10^{-4}					
**PPL Deaerated Solutions (*WCA 2A-O PPL*)**											
Arc 1		2474.9	**0.65**								
Arc 2		2482.2	**0.65**								
G1	Exocyclic S	2473.0	**0.63**	2.16	48						
G2	Heterocyclic S	**2474.3**	**0.63**	1.16	26						
G3	Sulfoxide	**2476.3**	**0.63**	0.03	0.7						
G4	Sulfone	**2479.5**	**0.84**	0.16	3.5						
G5	Sulfonate	**2481.2**	**0.84**	0.78	17						
G6	Organosulfate	**2482.8**	**0.84**	0.22	4.9						
NSS[d] = 4.2×10^{-4}											

[a] values in bold were co-varied and values in bold and underlined were fixed during Gaussian curve fitting.
[b] calculated using generic calibration curve.[10]
[c] precisions of atomic fractions are estimated to be ≤ 1.6%; the accuracy of the atomic fraction of each reduced and oxidized sulfur functionality is estimated at 8% and 4%, respectively.[10]
[d] the normalized sum-squared residual (NSS) is the normalized difference between experimental and fit spectra.

Table S2. Comparison of the elemental composition and relative abundance of sulfur species for a DOM sample isolated by three different solid-phase extraction (SPE) procedures. Sulfur functionalities include exocyclic reduced sulfur (DOS_{Exo}), heterocyclic reduced sulfur (DOS_{Hetero}), sulfoxide (DOS_{Sulfx}), sulfone (DOS_{SO2}), sulfonate (DOS_{SO3}), and organosulfate (DOS_{SO4}). Parameter values for Gaussian curve fitting of spectra can be found in **Table S1**.

				**Sulfur atomic fractions (f_{DOS_X}) measured by S K-edge XANES**						
**SPE Treatment**	**Carbon (mol/g)**	**Sulfur (mol/g)**	**Atomic S/C**	**DOS_{Exo} (%)**	**DOS_{Hetero} (%)**	**DOS_{Sulfx} (%)**	**DOS_{SO2} (%)**	**DOS_{SO3} (%)**	**DOS_{SO4} (%)**	**DOS_{Red}[a] (%)**
XAD-8 Ambient Atmosphere (*IS17-0004FL HPOA*)	4.19	0.040	$9.5x10^{-3}$3	50	28	1.0	2.7	15	4.1	78
XAD-8 Deaerated Solutions (*IS17-0005FL HPOA*)	3.93	0.040	$10.1x10^{-3}$	48	27	0.9	3.0	16	5.3	75
PPL Deaerated Solutions (*WCA 2A-O PPL*)	4.45	0.036	$8.1x10^{-3}$	48	26	0.7	3.5	17	4.9	74

[a] Total reduced sulfur is defined as the summation of exocyclic reduced (DOS_{Exo}) and heterocyclic reduced sulfur (DOS_{Hetero}).

Table S3. Parameter values for Gaussian curve fitting (GCF) and sulfur atomic fractions (%) of measured spectra of the hydrophobic organic acid (HPOA) fraction of DOM from laboratory oxidation experiments. GCF curves are shown in **Figure S6**.

**Function**	**S Functionality**	**Energy (eV)[a]**	**Width[a]**	**Calib. Area[b]**	**DOS Atomic Fraction (f_{DOS_X})[c] (%)**	**Function**	**S Functionality**	**Energy (eV)[a]**	**Width[a]**	**Calib. Area[b]**	**DOS Atomic Fraction (f_{DOS_X})[c] (%)**
**Oxidation Exp – t = 0 initial (*IS17-0006FL HPOA*)**						**Oxidation Exp – Dark, Anoxic Control (*IS17-0014FL HPOA*)**					
Arc 1		2475.2	**0.65**			Arc 1		2475.1	**0.64**		
Arc 2		2482.2	**0.65**			Arc 2		2482.4	**0.64**		
G1	Exocyclic S	2472.9	**0.66**	2.43	53	G1	Exocyclic S	2473.0	**0.64**	2.23	48
G2	Heterocyclic S	**<u>2474.4</u>**	**0.66**	1.34	29	G2	Heterocyclic S	**<u>2474.3</u>**	**0.64**	1.33	29
G3	Sulfoxide	**<u>2476.3</u>**	**0.66**	0.04	0.8	G3	Sulfoxide	**<u>2476.3</u>**	**0.64**	0.07	1.5
G4	Sulfone	**<u>2479.5</u>**	**0.86**	0.11	2.4	G4	Sulfone	**<u>2479.5</u>**	**0.84**	0.13	2.8
G5	Sulfonate	**<u>2481.2</u>**	**0.86**	0.56	12	G5	Sulfonate	**<u>2481.2</u>**	**0.84**	0.59	13
G6	Organosulfate	**<u>2482.8</u>**	**0.86**	0.14	2.9	G6	Organosulfate	**<u>2482.8</u>**	**0.84**	0.27	5.8
NSS[d] = 3.4 x 10^{-4}						*NSS*[d] = 3.8 x 10^{-4}					
**Oxidation Exp – Dark O2 (8 d) (*IS17-0007FL HPOA*)**											
Arc 1		2474.9	**0.66**								
Arc 2		2481.9	**0.66**								
G1	Exocyclic S	2473.0	**0.63**	2.22	50						
G2	Heterocyclic S	**<u>2474.3</u>**	**0.63**	1.31	30						
G3	Sulfoxide	**<u>2476.3</u>**	**0.63**	0.05	1.2						
G4	Sulfone	**<u>2479.5</u>**	**0.84**	0.12	2.7						
G5	Sulfonate	**<u>2481.2</u>**	**0.84**	0.59	13						
G6	Organosulfate	**<u>2482.8</u>**	**0.84**	0.16	3.5						
NSS[d] = 4.4 x 10^{-4}											

[a] values in bold were co-varied and values in bold and underlined were fixed during Gaussian curve fitting.
[b] calculated using generic calibration curve.[10]
[c] precisions of atomic fractions are estimated to be ≤ 1.6%; the accuracy of the atomic fraction of each reduced and oxidized sulfur functionality is estimated at 8% and 4%, respectively.[10]
[d] the normalized sum-squared residual (NSS) is the normalized difference between experimental and fit spectra.

Table S4. Parameter values for Gaussian curve fitting (GCF) and sulfur atomic fractions (%) of measured spectra of the hydrophobic organic acid (HPOA) fraction of DOM from laboratory Light treatments (GCF curves in **Figure S8**).

Function	S Functionality	Energy (eV)[a]	Width[a]	Calib. Area[b]	DOS Atomic Fraction ($f_{\mathrm{DOS_X}}$)[c] (%)	Function	S Functionality	Energy (eV)[a]	Width[a]	Calib. Area[b]	DOS Atomic Fraction ($f_{\mathrm{DOS_X}}$)[c] (%)
**Oxidation Exp – Light 1 (*IS17-0012FL HPOA*)**						**Oxidation Exp – Light 2 (*IS17-0011FL HPOA*)**					
Arc 1		2475.0	**0.65**			Arc 1		2475.1	**0.58**		
Arc 2		2482.1	**0.65**			Arc 2		2482.0	**0.58**		
G1	Exocyclic S	2473.0	**0.63**	2.06	47	G1	Exocyclic S	2473.0	**0.63**	1.88	44
G2	Heterocyclic S	**2474.4**	**0.63**	1.29	29	G2	Heterocyclic S	**2474.4**	**0.63**	1.27	30
G3	Sulfoxide	**2476.3**	**0.63**	0.08	1.8	G3	Sulfoxide	**2476.3**	**0.63**	0.09	2.2
G4	Sulfone	**2479.5**	**0.85**	0.13	2.9	G4	Sulfone	**2479.5**	**0.85**	0.14	3.3
G5	Sulfonate	**2481.2**	**0.85**	0.60	14	G5	Sulfonate	**2481.2**	**0.85**	0.64	15
G6	Organosulfate	**2482.8**	**0.85**	0.22	5.1	G6	Organosulfate	**2482.8**	**0.85**	0.27	6.2
NSS[d] = 3.5 x 10^{-4}						*NSS*[d] = 3.2 x 10^{-4}					
**Oxidation Exp – Light 3-1 (*IS17-0009FL HPOA*)**						**Oxidation Exp – Light 3-2 (*IS17-0015FL HPOA*)**					
Arc 1		2475.1	**0.65**			Arc 1		2475.2	**0.61**		
Arc 2		2482.1	**0.65**			Arc 2		2482.2	**0.61**		
G1	Exocyclic S	2473.1	**0.61**	1.60	39	G1	Exocyclic S	2473.1	**0.605**	1.65	39
G2	Heterocyclic S	**2474.4**	**0.61**	1.16	28	G2	Heterocyclic S	**2474.4**	**0.605**	1.26	30
G3	Sulfoxide	**2476.3**	**0.61**	0.13	3.2	G3	Sulfoxide	**2476.3**	**0.605**	0.15	3.5
G4	Sulfone	**2479.6**	**0.85**	0.15	3.7	G4	Sulfone	**2479.5**	**0.85**	0.14	3.4
G5	Sulfonate	**2481.2**	**0.85**	0.72	18	G5	Sulfonate	**2481.2**	**0.85**	0.74	18
G6	Organosulfate	**2482.8**	**0.85**	0.32	7.9	G6	Organosulfate	**2482.8**	**0.85**	0.25	6.0
NSS[d] = 2.9 x 10^{-4}						*NSS*[d] = 2.0 x 10^{-4}					
**Oxidation Exp – Light 4 (*IS17-0010FL HPOA*)**						**Oxidation Exp – Light 5 (*IS17-0008FL HPOA*)**					
Arc 1		2475.0	**0.57**			Arc 1		2475.2	**0.64**		
Arc 2		2481.9	**0.57**			Arc 2		2482.0	**0.64**		
G1	Exocyclic S	2473.1	**0.60**	1.28	32	G1	Exocyclic S	2473.1	**0.63**	1.16	27
G2	Heterocyclic S	**2474.4**	**0.60**	1.07	26	G2	Heterocyclic S	**2474.4**	**0.63**	0.97	23
G3	Sulfoxide	**2476.3**	**0.60**	0.13	3.1	G3	Sulfoxide	**2476.3**	**0.63**	0.16	3.7
G4	Sulfone	**2479.6**	**0.81**	0.20	4.9	G4	Sulfone	**2479.5**	**0.85**	0.24	5.6
G5	Sulfonate	**2481.2**	**0.81**	0.84	21	G5	Sulfonate	**2481.2**	**0.85**	1.19	28
G6	Organosulfate	**2482.8**	**0.81**	0.55	14	G6	Organosulfate	**2482.8**	**0.85**	0.57	13
NSS[d] = 2.0 x 10^{-4}											

[a] values in bold were co-varied and values in bold and underlined were fixed during Gaussian curve fitting.
[b] calculated using generic calibration curve.[10]
[c] precisions of atomic fractions are estimated to be ≤ 1.6%; the accuracy of the atomic fraction of each reduced and oxidized sulfur functionality is estimated at 8% and 4%, respectively.[10]
[d] The normalized sum-squared residual (NSS) is the normalized difference between experimental and fit spectra.

References

(1) Poulin, B. A.; Ryan, J. N.; Nagy, K. L.; Stubbins, A.; Dittmar, T.; Orem, W.; Krabbenhoft, D. P.; Aiken, G. R. Spatial Dependence of Reduced Sulfur in Everglades Dissolved Organic Matter Controlled by Sulfate Enrichment. *Environ. Sci. Technol.* **2017**, *51* (7), 3630–3639. https://doi.org/10.1021/acs.est.6b04142.

(2) Tate, M. T.; DeWild, J. F.; Ogorek, J. M.; Janssen, S. E.; Krabbenhoft, D. P.; Poulin, B. A.; Breitmeyer, S. E.; Aiken, G. R.; Orem, W. H.; Varonka, M. S. Chemical Characterization of Water, Sediments, and Fish from Water Conservation Areas and Canals of the Florida Everglades (USA), 2012 to 2019. *U.S. Geological Survey data release* **2023**. https://doi.org/10.5066/P976EGIX.

(3) Dittmar, T.; Koch, B.; Hertkorn, N.; Kattner, G. A Simple and Efficient Method for the Solid-Phase Extraction of Dissolved Organic Matter (SPE-DOM) from Seawater. *Limnol. Oceanogr. Methods* **2008**, *6* (6), 230–235. https://doi.org/10.4319/lom.2008.6.230.

(4) Ossola, R.; Tolu, J.; Clerc, B.; Erickson, P. R.; Winkel, L. H. E.; McNeill, K. Photochemical Production of Sulfate and Methanesulfonic Acid from Dissolved Organic Sulfur. *Environ. Sci. Technol.* **2019**, *53* (22), 13191–13200. https://doi.org/10.1021/acs.est.9b04721.

(5) Aiken, G. R.; McKnight, D. M.; Thorn, K. A.; Thurman, E. M. Isolation of Hydrophilic Organic Acids from Water Using Nonionic Macroporous Resins. *Org. Geochem.* **1992**, *18* (4), 567–573. https://doi.org/10.1016/0146-6380(92)90119-I.

(6) Weishaar, J. L.; Aiken, G. R.; Bergamaschi, B. a; Fram, M. S.; Fujii, R.; Mopper, K. Evaluation of Specific Ultraviolet Absorbance as an Indicator of the Chemical Composition and Reactivity of Dissolved Organic Carbon. *Environ. Sci. Technol.* **2003**, *37* (20), 4702–4708. https://doi.org/10.1021/es030360x.

(7) Helms, J. R.; Stubbins, A.; Ritchie, J. D.; Minor, E. C.; Kieber, D. J.; Mopper, K. Absorption Spectral Slopes and Slope Ratios as Indicators of Molecular Weight, Source, and Photobleaching of Chromophoric Dissolved Organic Matter. *Limnol. Oceanogr.* **2008**, *53* (3), 955–969. https://doi.org/10.4319/lo.2008.53.3.0955.

(8) Xu, Y.; Schoonen, M. A. A. The Stability of Thiosulfate in the Presence of Pyrite in Low-Temperature Aqueous Solutions. *Geochim. Cosmochim. Acta* **1995**, *59* (22), 4605–4622. https://doi.org/10.1016/0016-7037(95)00331-2.

(9) Ravel, B.; Newville, M. ATHENA, ARTEMIS, HEPHAESTUS: Data Analysis for X-Ray Absorption Spectroscopy Using IFEFFIT. *J. Synchrotron Radiat.* **2005**, *12* (Pt 4), 537–541. https://doi.org/10.1107/S0909049505012719.

(10) Manceau, A.; Nagy, K. L. Quantitative Analysis of Sulfur Functional Groups in Natural Organic Matter by XANES Spectroscopy. *Geochim. Cosmochim. Acta* **2012**, *99*, 206–223. https://doi.org/10.1016/j.gca.2012.09.033.

(11) Ksionzek, K. B.; Lechtenfeld, O. J.; McCallister, S. L.; Schmitt-Kopplin, P.; Geuer, J. K.; Geibert, W.; Koch, B. P. Dissolved Organic Sulfur in the Ocean: Biogeochemistry of a Petagram Inventory. *Science* **2016**, *354* (6311), 456–459. https://doi.org/10.1126/science.aaf7796.

(12) Gomez-Saez, G. V; Pohlabeln, A. M.; Stubbins, A.; Marsay, C. M.; Dittmar, T. Photochemical Alteration of Dissolved Organic Sulfur from Sulfidic Porewater. *Environ. Sci. Technol.* **2017**, *51* (24), 14144–14154. https://doi.org/10.1021/acs.est.7b03713.
